# Supplementary material for: Topological prethermal strong zero modes on superconducting processors
Source: Nature. 2025 Aug 27;645(8081):626–32. doi: 10.1038/s41586-025-09476-z (PMC12443636; doi:10.1038/s41586-025-09476-z)
Supplement: Supplementary file 1 — Supplementary Information [file 41586_2025_9476_MOESM1_ESM.pdf]

nature portfolio

https://doi.org/10.1038/s41586-025-09476-z

Supplementary information

Topological prethermal strong zero modes on superconducting processors

In the format provided by the authors and unedited

Supplementary Information for
"Topological prethermal strong zero modes on superconducting processors"

CONTENTS

1. Theoretical analysis S1
   A. Overall pictures and relevant timescales S1
   B. Floquet prethermalization and effective Hamiltonian S2
   C. 1D SPT spin chain and edge modes at zero temperature S4
   D. Edge modes as prethermal strong zero modes at finite temperatures S4
   E. Prethermalization due to dimerization and emergent U(1)×U(1) symmetry S5
   F. Jordan-Wigner transformation and Majorana fermion picture S6
   G. Energy spectroscopy in an integrable chain S8
   H. Noiseless simulation via matrix product states S10

2. Experimental information S11
   A. Device performance S11
   B. Gate calibration S11
   C. Experimental circuits S12
   D. Mitigation of leakage error S12
   E. Quantum state tomography S15
   F. Comparison of noise resilience between edge modes and physical qubits S16
   G. More data for echo experiments S17

   References S17

1. THEORETICAL ANALYSIS

A. Overall pictures and relevant timescales

We first outline the overall picture of the time dynamics and relevant timescales in our work (Fig. S1**a**). Our model involves two distinct prethermal mechanisms: Floquet prethermalization (governed by Trotter step $J\delta t$) and the prethermalization via dimerization (controlled by parameters $J_{\mathrm{o}}, J_{\mathrm{e}}$). The former establishes a regime well-described by an effective time-independent Hamiltonian $H_{\mathrm{F}} \approx H_0 + H_1$ and preserves energies until a late heating time t_*. The latter gives an emergent U(1)×U(1) symmetry with a lifetime t_{sym}, which underpins the stability of the prethermal strong zero modes. In our experiments, we primarily focus on the second mechanism, selecting $J\delta t$ such that t_* is far beyond the experimental timescales t_{exp}. Consequently, the system dynamics are effectively described by H_{F}, under which two factors bound the edge mode lifetime τ: The hybridization between edge modes at each end of the chain, and the interaction between edge modes and thermal excitations in the bulk (Fig. S1**b**). The former depends on the system size N, leading to a lifetime t_{N} scaling exponentially with N. The latter depends on both the effective temperature of initial states and the dimerization strength $J_{\mathrm{o}}/J_{\mathrm{e}}$. When $J_{\mathrm{o}}/J_{\mathrm{e}}$ is near resonant points, thermal excitations in the bulk can interact strongly with the edge modes and result in their rapid decay. As $J_{\mathrm{o}}/J_{\mathrm{e}}$ moves away from resonances, the prethermalization gives rise to the emergent U(1)×U(1) symmetry, prolonging τ to the symmetry lifetime t_{sym} (Fig. S1**c**). In finite-sized systems, τ is ultimately upper bounded by t_{N} (Fig. S1**d**).

In the following subsections, we first analyze the Floquet prethermalization in our Trotterized circuits and derive the effective Hamiltonian H_{F} (Sec. 1B). We then focus on $H_{\mathrm{F}} \approx H_0 + H_1$, analyzing 1D symmetry-protected topological (SPT) spin Hamiltonian and the corresponding edge modes at zero temperature (Sec. 1C). We explain why thermal excitations ruin it at finite temperatures, and how it presents as prethermal strong zero modes with dimerized parameters in the system Hamiltonian (Sec. 1D). Such strong zero modes can be understood as a consequence of U(1)×U(1) symmetry, which is approximately conserved by prethermalization due to dimerization (Sec. 1E). We introduce how to map the spin Hamiltonian into two Kitaev chains by applying the Jordan-Wigner transformation (Sec. 1F). We explain how energy spectroscopy is carried out and the form of energy gaps in an integrable chain (Sec. 1G). Finally, we provide the noiseless simulation results for our system via matrix-product states (Sec. 1H).

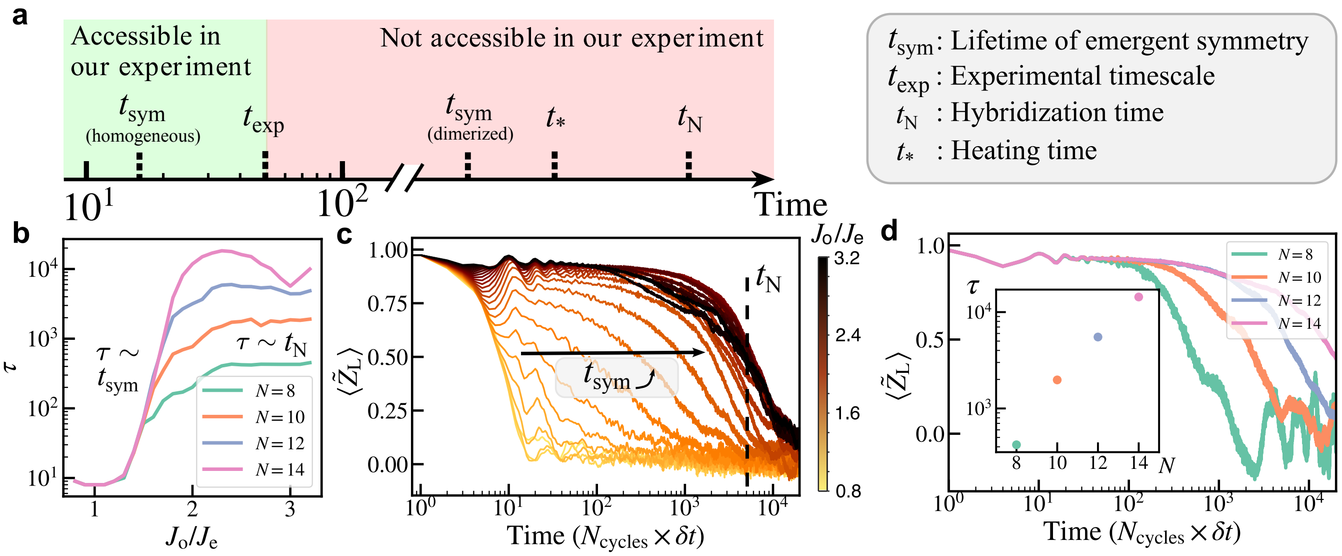

Fig. S1. **a**, Timescales relevant to our experiments. **b**, the edge mode lifetime τ as a function of N and J_o/J_e, presenting two different scaling regimes $\tau \sim t_{\rm sym}$ and $\tau \sim t_{\rm N}$. **c**, In a chain with $N = 12$ qubits and J_o/J_e close to resonance points, τ is bounded by interactions with bulk excitations. As J_o/J_e deviates from the resonant points, the interaction is suppressed by the emergent U(1)×U(1) symmetry in the prethermal regime, and $\tau \sim t_{\rm sym}$ increases. τ eventually saturates to a finite value $t_{\rm N}$ due to the hybridization between two edge modes in the finite-sized system. **d**, In the dimerized regime with $J_o/J_e = 2.618$ where edge-bulk interactions being sufficiently suppressed, $\tau \sim t_{\rm N}$ scales nearly exponentially with N. All the data are obtained by noiseless simulation with Trotter evolution $U(\delta t) = U_1(\delta t)U_0(\delta t)$, effectively infinite-temperature initial state $|00\ldots0\rangle$, and the parameters being the same as the ones in the main text ($\delta t = 0.5, J_e = \pi/5, h_x = 0.11, V_{xx} = 0.2$).

B. Floquet prethermalization and effective Hamiltonian

In our experiments, we aim to implement the evolution governed by the time-independent Hamiltonian $H = H_0(J_e, J_o) + H_1(h_x, V_{xx})$, where H_0 and H_1 read

$$H_0(J_e, J_o) = J_e \sum_{i=1}^{N/2-1} \sigma^z_{2i-1}\sigma^x_{2i}\sigma^z_{2i+1} + J_o \sum_{i=1}^{N/2-1} \sigma^z_{2i}\sigma^x_{2i+1}\sigma^z_{2i+2}, \tag{S1}$$

$$H_1(h_x, V_{xx}) = h_x \sum_{i=1}^{N} \sigma^x_i + V_{xx} \sum_{i=1}^{N-1} \sigma^x_i\sigma^x_{i+1}. \tag{S2}$$

Such a Hamiltonian is suitable for theoretical analysis, yet poses considerable challenges for experimental implementation. This is due to the three-body interactions inherent in H_0, and the fact that H_0 and H_1 do not commute. We digitally simulate the evolution of H_0, H_1 within a time step δt by quantum circuits $U_0(\delta t)$, $U_1(\delta t)$. The evolution of $H_0 + H_1$ is then approximated by first-order Trotter decomposition $U(\delta t) = U_1(\delta t)U_0(\delta t)$. This is a time-dependent Floquet evolution, under which the energy is no longer conserved, and the system will ultimately thermalize to infinite temperature. However, under high-frequency drives (and hence small Trotter steps $J\delta t$ in our case), the system will first relax to a metastable state with almost conserved energies until a heating time t_* that is exponential in $1/(J\delta t)$. This process is known as the Floquet prethermalization [S1–S3]. We verify this energy conservation by numerical simulations and show the results in Fig. S2**a**. It is clear that the system heating rate measured by $|E(t)/E(t=0)|$ is slowed down with decreased Trotter step size (light curves, $\delta t = 0.5, 0.1$). However, the lifetime of neither edge modes nor U(1)×U(1) symmetry is enlarged, indicating that the Floquet prethermalization does not give rise to the U(1)×U(1) symmetry in our work, and hence is not the origin of the robust edge modes.

Another striking property of the Floquet prethermalization is that, within the heating time t_*, the system's evolution at stroboscopic time $t = n\delta t$ can be described by a time-independent effective Hamiltonian H_F as $U(n\delta t) = \exp[-i(n\delta t)H_F]$. Such an effective Hamiltonian is obtained from applying Floquet-Magnus expansion [S4–S6] order by order:

$$H_F = \sum_{n=0}^{\infty} (\delta t)^n \Omega_n. \tag{S3}$$

This series expansion typically does not converge, potentially signaling the ergodicity of the Floquet process. However, given the scaling of the coefficient of the n-th order term proportional to $(\delta t)^n$, we expect that higher-order terms will not take effect

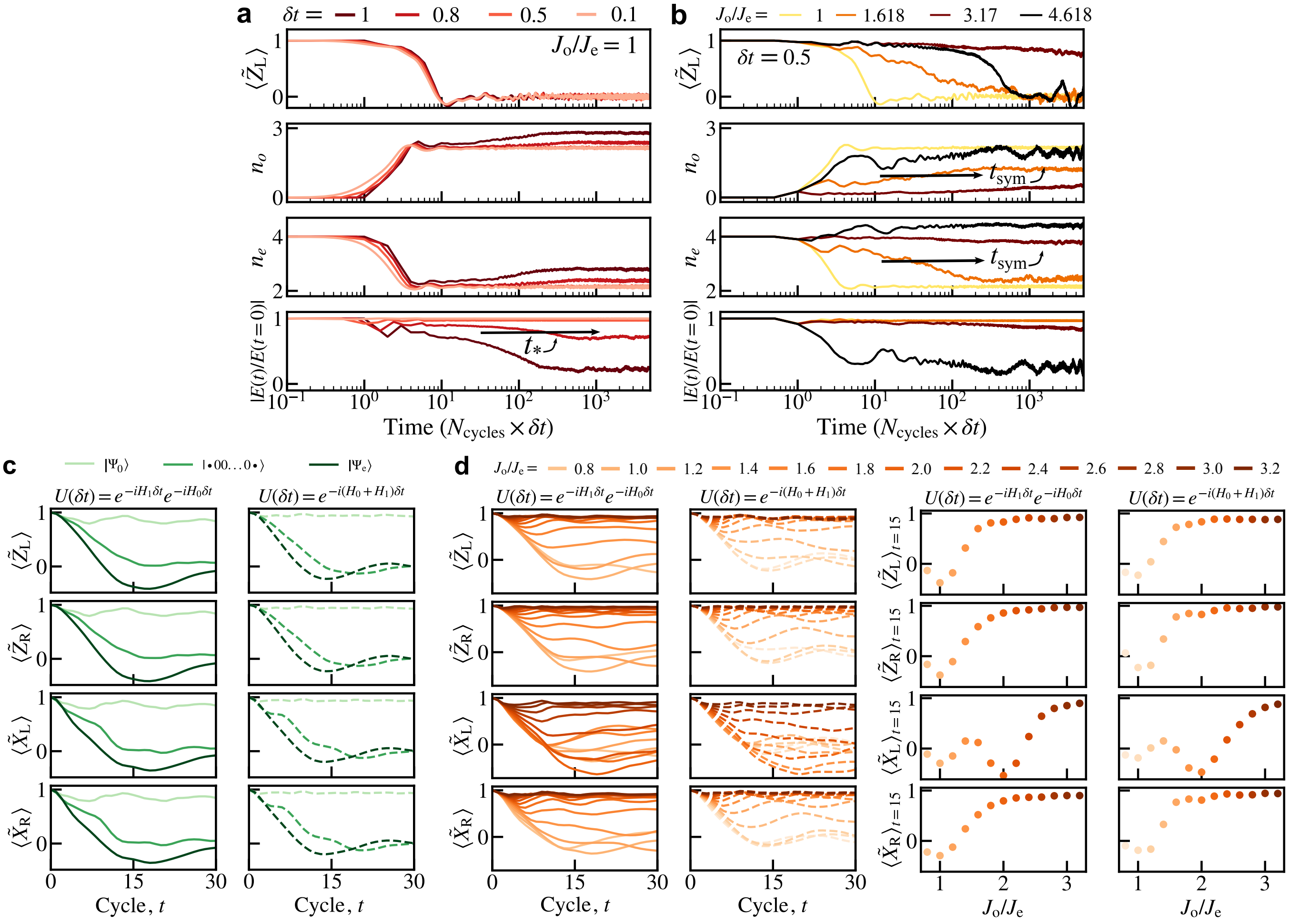

Fig. S2. **a**, Short-step Trotterized evolutions induce the Floquet prethermal regime with effective Hamiltonian and almost conserved energy (bottom panel, light curves), while the edge modes and U(1)×U(1) symmetry are not conserved. **b**, The derivation of J_o/J_e from the resonant point induces the prethermal regime with the emergent U(1)×U(1) symmetry and long-lived edge modes. **c,** Measured time dynamics for edge operators under the Trotter circuit (left) and the exact evolution (right) in the homogeneous regime ($J_o = J_e = \pi/5$). **d,** Measured time dynamics for edge operators with fixed $J_e = \pi/5$ and varying J_o. Data in the first (second) and third (fourth) columns are obtained from the Trotter circuit (exact evolution). The numerical calculations are carried out on a 14-qubit system with $h_x = 0.11$ and $V_{xx} = 0.2$. The initial states in **a, b, d** and the $|\Psi_e\rangle$ in **c** are the cluster states with 4 excitations.

until a considerable delayed time, provided that δt is relatively small. Consequently, truncating the series expansion after the first few orders usually suffices to depict the system behavior within an experimental timescale. In our scenario, the first two orders read,

$$\Omega_0 = \frac{1}{\delta t}\int_0^{\delta t} H(t_1)\mathrm{d}t_1 = H_0 + H_1, \tag{S4}$$

$$\begin{aligned}
\Omega_1 &= \frac{1}{2i(\delta t)^2}\int_0^{\delta t}\mathrm{d}t_1\int_0^{t_1}\mathrm{d}t_2[H(t_1),H(t_2)] \\
&= J_e h_x \sum_{i=1}^{N/2-1}\left(\sigma^y_{2i-1}\sigma^x_{2i}\sigma^z_{2i+1} + \sigma^z_{2i-1}\sigma^x_{2i}\sigma^y_{2i+1}\right) + J_o h_x \sum_{i=1}^{N/2-1}\left(\sigma^y_{2i}\sigma^x_{2i+1}\sigma^z_{2i+2} + \sigma^y_{2i}\sigma^x_{2i+1}\sigma^y_{2i+2}\right) \\
&\quad + J_e V_{xx}\left[\sum_{i=2}^{N/2-1}\sigma^x_{2i-2}\sigma^y_{2i-1}\sigma^x_{2i}\sigma^z_{2i+1} + \sum_{i=1}^{N/2-1}\left(\sigma^y_{2i-1}\sigma^z_{2i+1} + \sigma^z_{2i-1}\sigma^y_{2i+1} + \sigma^z_{2i-1}\sigma^x_{2i}\sigma^y_{2i+1}\sigma^x_{2i+2}\right)\right] \\
&\quad + J_o V_{xx}\left[\sum_{i=1}^{N/2-1}\left(\sigma^x_{2i-1}\sigma^y_{2i}\sigma^x_{2i+1}\sigma^z_{2i+2} + \sigma^y_{2i}\sigma^z_{2i+2} + \sigma^z_{2i}\sigma^y_{2i+2}\right) + \sum_{i=1}^{N/2-2}\sigma^z_{2i}\sigma^x_{2i+1}\sigma^y_{2i+2}\sigma^x_{2i+3}\right].
\end{aligned} \tag{S5}$$

This gives $H_{\mathrm{F}}^{(2)} = H_0 + H_1 + (\delta t)\Omega_1$. Considering higher-order expansions introduces additional terms, leading to more general interactions between the edges and the bulk. The prethermal strong zero modes are quite robust and can be observed in the presence of these Trotter errors. As shown in Fig. S2**c-d**, we carry out the noiseless simulation on the temporal dependence of the edge modes under the Trottered circuit $e^{-iH_1\delta t}e^{-iH_0\delta t}$ and the exact evolution $e^{-i(H_0+H_1)\delta t}$. We observe that the edge mode behavior is qualitatively similar in both scenarios: they decay rapidly when initialized in finite-temperature states but show resilience against excitations with dimerized $J_{\mathrm{o}}/J_{\mathrm{e}}$. Strikingly, the Trotter errors do not change the first-order resonance points at $J_{\mathrm{o}}/J_{\mathrm{e}} = 1$ and 2. Therefore, in the following, we will focus on analyzing the behavior of PSZMs with respect to the first-order expansion $H_{\mathrm{F}}^{(1)} = H_0 + H_1$.

C. 1D SPT spin chain and edge modes at zero temperature

Our 1D Hamiltonian $H_{\mathrm{F}}^{(1)} = H_0 + H_1$ comprises two ingredients: The first part H_0 includes strong interaction among neighboring sites, which introduces SPT phases in our system. The second part H_1 considers perturbation terms, which include a transverse field in x direction and two-body XX interactions for neighboring qubits. We first investigate the properties of the SPT Hamiltonian H_0 shown in Eq. (S1), which contains N qubits in total. The Hamiltonian preserves a $\mathbb{Z}_2\times\mathbb{Z}_2$ symmetry, which is generated by the parity operators on even sites $G_{\mathrm{e}} = \prod_{i=1}^{N/2}\sigma_{2i}^x$ and odd sites $G_{\mathrm{o}} = \prod_{i=1}^{N/2}\sigma_{2i-1}^x$. The three-body interacting terms $K_i = \sigma_{i-1}^z\sigma_i^x\sigma_{i+1}^z$ in H_0 commutes with each others and are called stabilizers. Note that there are only $N-2$ stabilizers in H_0, while the system degree of freedom is N. This leads to the system having four-fold degenerate manifolds, both at the bottom and top of the spectrum, where stabilizers all equal to -1 or $+1$. Within each of these manifolds, the degenerated states can be distinguished by two edge modes induced by the $\mathbb{Z}_2\times\mathbb{Z}_2$ symmetry. To see this, first note that the parity operators can be decomposed into the product of stabilizers and operators at edges:

$$G_{\mathrm{e}} = \sigma_1^z\left(\prod_{i=1}^{N/2-1} K_{2i}\right)\sigma_{N-1}^z\sigma_N^x, \quad G_{\mathrm{o}} = \sigma_1^x\sigma_2^z\left(\prod_{i=1}^{N/2-1} K_{2i+1}\right)\sigma_N^z. \tag{S6}$$

With all K_i equal to either -1 or 1, these parity operators can be further projected onto the edges. For example, in our experiment with all K_i taking 1, these parity operators read,

$$G_{\mathrm{e}} = \sigma_1^z\left(\sigma_{N-1}^z\sigma_N^x\right) = \tilde{Z}_{\mathrm{L}}\tilde{X}_{\mathrm{R}}, \quad G_{\mathrm{o}} = \left(\sigma_1^x\sigma_2^z\right)\sigma_N^z = \tilde{X}_{\mathrm{L}}\tilde{Z}_{\mathrm{R}}, \quad \begin{cases}\tilde{Z}_{\mathrm{L}} \equiv \sigma_1^z \\ \tilde{X}_{\mathrm{L}} \equiv \sigma_1^x\sigma_2^z\end{cases}, \quad \begin{cases}\tilde{Z}_{\mathrm{R}} \equiv \sigma_N^z \\ \tilde{X}_{\mathrm{R}} \equiv \sigma_{N-1}^z\sigma_N^x\end{cases}. \tag{S7}$$

As H_0 is local, the preserved symmetry, $[H_0, G_{\mathrm{e}}] = [H_0, \tilde{Z}_{\mathrm{L}}\tilde{X}_{\mathrm{R}}] = 0$, gives rise to both $[H_0, \tilde{Z}_{\mathrm{L}}] = 0$ and $[H_0, \tilde{X}_{\mathrm{R}}] = 0$. Similar results are obtained from $[H_0, G_{\mathrm{o}}] = 0$. They together give four conserved quantities $\tilde{Z}_{\mathrm{L}}$, $\tilde{X}_{\mathrm{L}}$, $\tilde{Z}_{\mathrm{R}}$, and $\tilde{X}_{\mathrm{R}}$ at the edges. In addition, since $\tilde{Z}$ and $\tilde{X}$ are anti-commuted at both left and right edges, the degeneracy is four-fold, with two effectively spin-1/2 edge modes described by $\tilde{Z}$ and $\tilde{X}$ residing at two ends of the chain. These edge operators connect the different sectors of the zero-temperature manifold.

When generic perturbations are added into H_0, the $\mathbb{Z}_2\times\mathbb{Z}_2$ symmetry is broken as the parity operators G_{e}, G_{o} are no longer preserved, destroying the edge modes. However, if the perturbations also preserve the $\mathbb{Z}_2\times\mathbb{Z}_2$ symmetry with considerably small strength compared with the strength of stabilizers in H_0, which is the case in our work, the state remains deep in the SPT phase, with the original localized edge modes now extending to the bulk. These extensions make the left and right edge modes hybridize with each other, resulting in the original degenerated states now opening energy gaps $\zeta \propto \exp(-N)$ exponentially small in the system size (which we observed in the main text Fig. 4 and Extended Data Fig. 7). This gives rise to the exponentially long lifetime t_{N} for edge modes at zero temperature under symmetry-preserved perturbations.

D. Edge modes as prethermal strong zero modes at finite temperatures

The above discussion is restricted to the system at zero temperature. At finite temperatures, thermal excitations emerge within the systems, interacting with edge modes and decohering them. The vulnerability of edge modes against thermal excitations is uncovered by the fact that $\left(\prod_{i=1}^{N/2-1}K_{2i}\right)$ and $\left(\prod_{i=1}^{N/2-1}K_{2i+1}\right)$ in Eq. (S6) are no longer conserved quantities for excited states, and hence G_{e}, G_{o} cannot be projected into the edges. For example, for the perturbation term H_1 in Eq. (S2), which contains single-body σ_i^x and two-body $\sigma_i^x\sigma_{i+1}^x$ perturbations, $\sigma_1^x\sigma_2^x$ or the second-order process of $\sigma_1^x + \sigma_2^x$ can flip $\tilde{Z}_{\mathrm{L}}$, $\tilde{X}_{\mathrm{L}}$, K_2, and K_3, while keeping G_{e} and G_{o} unchanged. If one of K_2 and K_3 is equal to -1, and both exhibit identical strength (i.e. $J_{\mathrm{e}} = J_{\mathrm{o}}$), this process further preserves the system energy and becomes a resonant perturbation, leading to rapid decoherence of both $\tilde{Z}_{\mathrm{L}}$ and $\tilde{X}_{\mathrm{L}}$. Physically, this represents transferring an excitation between the even and odd sites through the edge (Fig. S3a). Notably, the first-order resonance can happen at edges even for unequal stabilizer strength. For example, in the main text, we observe the

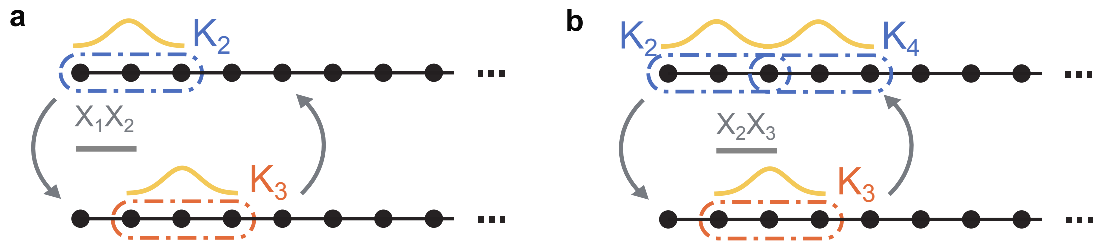

Fig. S3. **First-order resonances.** **a**, In the system with homogeneous stabilizer strength $J_o = J_e$, the two-body interaction $V_{xx}\sigma_1^x\sigma_2^x$ in H_1 can resonantly exchange one excitation between J_eK_2 and J_oK_3, which also flips $\tilde{Z}_L$ and $\tilde{X}_L$. **b**, In the system with stabilizer strength $J_o = 2J_e$, the $V_{xx}\sigma_2^x\sigma_3^x$ term resonantly transfers two excitations J_eK_2, J_eK_4 into J_oK_3, and vice versa. This process makes $\tilde{X}_L$ rapidly decohered. A similar process can happen at the right edge with $J_o = 0.5J_e$ and is not shown.

left edge operator $\tilde{X}_L$ is rapidly decohered when $J_o/J_e = 2$. This is caused by the $\sigma_2^x\sigma_3^x$ term which flips $\tilde{X}_L$, K_2, K_3, and K_4, and hence resonantly transferring two excitations with energy J_e to one excitation with energy J_o (Fig. S3b). Similar process can happen for $\tilde{X}_R$ at right edge when $J_o/J_e = 0.5$. For systems taking other dimerized stabilizer strengths, the resonance only occurs in higher-order processes, which are hard to observe within the current experimental timescale.

Theoretically, a local operator being approximately conserved for arbitrary system configurations is characterized by a prethermal strong zero mode (PSZM) [S7–S9]. Such an operator almost commutes with the system Hamiltonian and maps the system eigenstates from one symmetry sector to another. By definition, a PSZM must satisfy the following three conditions: (1) Squares to the identity. (2) Almost commutes with the Hamiltonian with exponentially small corrections in the system size. (3) Anti-commutes with the system's symmetry. In practice, such an operator is constructed from perturbation theory order by order and is cut off at some finite order to obtain a bounded commutator with the Hamiltonian. In our setting, the PSZM can be constructed for all $\tilde{Z}_L$, $\tilde{X}_L$, $\tilde{Z}_R$, and $\tilde{X}_R$, which leads to robust edge modes locally encoding spin-1/2 degrees of freedom at both ends of the chain at finite temperatures. Up to the first order in h_x and V_{xx}, such PSZMs for the left edge reads [S9],

$$\Psi_L^z = \tilde{Z}_L + \frac{h_x}{J_e}\sigma_1^x\sigma_2^x\sigma_3^z - \frac{V_{xx}}{J_o^2 - J_e^2}(J_e\sigma_1^x\sigma_3^z + J_o\sigma_1^y\sigma_2^y\sigma_3^x\sigma_4^z), \tag{S8}$$

$$\begin{aligned}\Psi_L^x = \tilde{X}_L &+ \frac{h_x}{J_o}\sigma_1^x\sigma_2^x\sigma_3^x\sigma_4^z + \frac{V_{xx}}{J_o^2 - J_e^2}(J_o\sigma_2^x\sigma_3^x\sigma_4^z + J_e\sigma_1^z\sigma_2^z\sigma_3^z) \\ &- \frac{V_{xx}J_e}{J_o^2 - 4J_e^2}\left[\sigma_1^y\sigma_2^z\sigma_3^y + \left(\frac{2J_e}{J_o} - \frac{J_o}{J_e}\right)\sigma_1^x\sigma_2^x\sigma_4^z - \sigma_1^x\sigma_2^y\sigma_3^y\sigma_4^x\sigma_5^z - \frac{2J_e}{J_o}\sigma_1^y\sigma_4^y\sigma_5^z\right],\end{aligned} \tag{S9}$$

and similar PSZMs can be constructed for the right edge. We identify that both Ψ_L^z and Ψ_L^x commute with $H_0 + H_1$ and squares to the identity up to error terms with order $O\left(\max\{h_x^2, V_{xx}^2\}\right)$. For a homogeneous system with $J_o = J_e$, the first-order terms in both Ψ_L^z, Ψ_R^x diverge, resulting from the resonant process of $V_{xx}\sigma_1^x\sigma_2^x$. In addition, we find Ψ_L^x also diverges at $J_o = 2J_e$, characterizing the effect of $V_{xx}\sigma_2^x\sigma_3^x$. These low-order resonances result in the dramatically reduced lifetime of the edge modes. The signs of coefficients change in some of bulk terms as we observed in Fig. 2 and Extended Data Fig. 4 of the main text. Besides these divergent points, Ψ_L^z, Ψ_R^x keep finite and satisfy $\{\Psi_L^z, G_o\} = [\Psi_L^z, G_e] = 0$ and $\{\Psi_L^x, G_e\} = [\Psi_L^x, G_o] = 0$. This induces almost conserved degeneracy throughout the entire spectrum and large overlaps between Ψ_L^z (Ψ_L^x) and $\tilde{Z}_L$ ($\tilde{X}_L$), which gives rise to robust and long-lived edge modes for arbitrary system configurations.

E. Prethermalization due to dimerization and emergent U(1)×U(1) symmetry

It was recently shown in Ref. [S10] that PSZMs arise in Hamiltonians with integer number spectra under small perturbations. The authors proved that there was an additional emergent U(1) symmetry present in the evolution of a Kitaev chain, given that the perturbations were much smaller than the interaction strength. This gave rise to robust Majorana edge modes at finite temperatures. For the SPT chain in our experiments, a similar U(1) symmetry is observed in the homogeneous regime (Fig. 3**d** in the main text), however, it is insufficient for protecting the spin-1/2 edge modes. Instead, we observe that the robust edge modes only occur under the protection of the U(1)×U(1) symmetry in the dimerized and off-resonant case.

We start by first investigating the emergent U(1) symmetry in the homogeneous case, which comes from considering the structure of the stabilizer terms in the Hamiltonian H. It has been shown that after applying local Schrieffer-Wolff transformations order by order and stopping at a certain order to restrict the growth of perturbation terms, the symmetry-breaking perturbations can be eliminated in a rotated frame. In particular, if the system Hamiltonian takes the form

$$H = JQ + V, \tag{S10}$$

with Q being a sum of mutually commuting local terms and having integer eigenvalues, and V being a sum of local perturbations with energy scale J_0, the evolution generated by H can be approximated by the following equation [S3, S11, S12]:

$$\exp(-iHt) = \mathcal{V}\exp\left[-i(JQ + V_p + E)t\right]\mathcal{V}^\dagger, \quad [Q, V_p] = 0, \quad \|E\| = O\left[\exp\left(-\frac{J}{J_0}\right)\right]. \tag{S11}$$

Note that the system now preserves Q up to an exponentially small error term E, indicating that an additional U(1) symmetry generated by Q emerges. The system will eventually lose this U(1) symmetry due to the errors E and equilibrate to the Gibbs state $e^{-\beta H}$ where β is determined by the initial state energy. However, before that, the effective Hamiltonian $JQ+V_p$ and the U(1) symmetry will survive for an exponentially long lifespan, referred to as the prethermal regime. In homogeneous system with $J_\mathrm{e} = J_\mathrm{o}$, the Hamiltonian $H = J_\mathrm{e}\sum_{i=2}^{N-1} K_i + H_1(h_x, V_{xx})$ exactly fits into Eq. (S10). Given that $J_\mathrm{e} \gg \max\{h_x, V_{xx}\}$, the sum of stabilizers in bulk $\sum_{i=2}^{N-1} K_i$ is approximately conserved. This leads to the conservation law on the total excitation number $(N-2-\sum_{i=2}^{N-1} K_i)/2$, which generates the U(1) symmetry in the prethermal regime lasting for a time scale of $O\left[\exp(J_\mathrm{e}/\max\{h_x, V_{xx}\})\right]$ time.

The edge modes fail to maintain robustness at finite temperatures, even in the presence of this additional U(1) symmetry. This is exemplified by the first-order resonant process depicted in Fig. S3**a**, where transferring an excitation between sites with different parity ruins the edge modes without modifying the total excitation number. The robust edge mode requires a larger symmetry group U(1)×U(1), denoting the conservation laws on excitation numbers within even and odd sites. Such a U(1)×U(1) symmetry can emerge within the system with dimerized stabilizer strength $J_\mathrm{e} \neq J_\mathrm{o}$ and not being at resonance. Intuitively, this separates the energy scales of stabilizers on the even and odd sites, leading to large energy obstacles to exchanging excitations. However, if the $J_\mathrm{e}/J_\mathrm{o}$ is a rational number, there could be resonant processes in the perturbation theory, which happens at a finite order independent with J_o, J_e and the system size. The first-order resonance in the $J_\mathrm{o} = 2J_\mathrm{e}$ case is an example (Fig. S3**b**). To avoid these resonances, the stabilizer strengths should be dimerized to be irrational multiples of each other. Then, the U(1)×U(1) symmetry is maintained within the exponentially long prethermal regime [S9, S13]. Formally, for the Hamiltonian taking the form

$$H = \sum_{i=1}^{m} J_i Q_i + V, \tag{S12}$$

with $\{Q_i\}$ being mutually commuting operators taking integer eigenvalues, and $\{J_i\}$ being irrational multiples of each other, and V being a sum of local perturbations with energy scale J_0, the evolution generated by H can be approximated by the following equation [S13]:

$$\exp(-iHt) = \mathcal{V}\exp\left[-i\left(\sum_{i=1}^{m} J_i Q_i + V_p + E\right)t\right]\mathcal{V}^\dagger, \quad \forall i,\ [Q_i, V_p] = 0, \quad \|E\| = O\left[\exp\left(-\left(\frac{|\boldsymbol{J}|}{J_0}\right)^{1/(m+\epsilon)}\right)\right], \tag{S13}$$

with $|\boldsymbol{J}|$ being the overall energy scale of $\{J_i\}$ and ϵ being a small constant. Within the rotated frame $\mathcal{V}$, the system now presents m approximate conservation laws on all Q_i, leading to the emergent $\mathrm{U}(1)^{\times m}$ symmetry. Note that for $m = 1$ the equation aligns with the one in Eq. (S11), and for our case with $m = 2$, the lifetime t_sym of the emergent U(1)×U(1) is exponential in the ratio of the energy scale of H_0 compared to the perturbation H_1. This aligns with our numerical results: In Fig S2**b**, we observe that both U(1) symmetries on the odd and even sites become approximately conserved as $J_\mathrm{o}/J_\mathrm{e}$ deviates from the first-order resonant point from 1 up to 3.17, leading to prolonged lifetime of the edge modes. Crucially, further increasing J_o to 4.618 will lead to the breakdown of Floquet prethermalization, resulting in rapid thermalization and again short lifetimes of edge mode and symmetry (dark curve). In our experiments, we bounded the parameter $J_\mathrm{o}/J_\mathrm{e}$ up to 3.2 to avoid the rapid heating, ensuring that heating only slightly affects the conservation laws on excitation numbers in the late time (Fig. S6c).

The key point to understand how this emergent U(1)×U(1) symmetry gives rise to the robust edge modes is that the conserved sum of stabilizers on even and odd sites will also lead to the conserved parity of stabilizers on even and odd sites, i.e. $\prod_{i=1}^{N/2-1} K_{2i}$ and $\prod_{i=1}^{N/2-1} K_{2i+1}$, which are originally not conserved when there are thermal excitations that can be exchanged resonantly. With these conserved parity operators, together with the system preserved $\mathbb{Z}_2 \times \mathbb{Z}_2$ symmetry, we can again project $G_\mathrm{e}, G_\mathrm{o}$ in Eq. (S6) into the edges and obtain Eq. (S7) without requiring the system being at zero temperature, leading to robust edge operators $\tilde{Z}, \tilde{X}$ for arbitrary system configurations.

F. Jordan-Wigner transformation and Majorana fermion picture

In the main text, we argue that our SPT qubit chain can be transformed into two Kitaev chains in the Majorana picture. To see this, we first consider applying the Jordan-Wigner transformation [S14] to map the spin Hamiltonian $H = H_0 + H_1$ into fermionic creation/annihilation operators $c_i^\dagger, c_i$:

$$\sigma_i^x = 1 - 2c_i^\dagger c_i, \quad \sigma_i^z = -\left[\prod_{j=1}^{i-1}(1 - 2c_j^\dagger c_j)\right](c_i^\dagger + c_i), \tag{S14}$$

and the inverse transformation is $c_i = -\frac{1}{2}\left(\prod_{j=1}^{i-1}\sigma_j^x\right)\sigma_i^z(1-\sigma_i^x)$, $c_i^\dagger = -\frac{1}{2}\left(\prod_{j=1}^{i-1}\sigma_j^x\right)(1-\sigma_i^x)\sigma_i^z$. This readily gives the canonical fermionic algebra $\{c_k, c_l^\dagger\} = \delta_{kl}, \{c_k, c_l\} = 0$. After applying Eq. (S14), H is transformed into the following form:

$$\begin{aligned}
H_\mathrm{f} = {} & J_\mathrm{e}\sum_{i=1}^{N/2-1}(c_{2i-1}^\dagger - c_{2i-1})(c_{2i+1}^\dagger + c_{2i+1}) + J_\mathrm{o}\sum_{i=1}^{N/2-1}(c_{2i}^\dagger - c_{2i})(c_{2i}^\dagger + c_{2i}) + h_x\sum_{i=1}^{N}(1-2c_i^\dagger c_i) \\
& + V_{xx}\sum_{i=1}^{N-1}(1-2c_i^\dagger c_i)(1-2c_{i+1}^\dagger c_{i+1}) \qquad \text{(S15)} \\
= {} & \underbrace{\sum_{i=1}^{N/2-1}\left[J_\mathrm{e}(c_{2i-1}^\dagger - c_{2i-1})(c_{2i+1}^\dagger + c_{2i+1}) + h_x(1-2c_{2i-1}^\dagger c_{2i-1})\right] + h_x(1-2c_{N-1}^\dagger c_{N-1})}_{\text{Upper Kitaev chain}} \\
& + \underbrace{\sum_{i=1}^{N/2-1}\left[J_\mathrm{o}(c_{2i}^\dagger - c_{2i})(c_{2i+2}^\dagger + c_{2i+2}) + h_x(1-2c_{2i}^\dagger c_{2i})\right] + h_x(1-2c_N^\dagger c_N)}_{\text{Lower Kitaev chain}} \\
& + \underbrace{V_{xx}\sum_{i=1}^{N-1}(1-2c_i^\dagger c_i)(1-2c_{i+1}^\dagger c_{i+1})}_{\text{Inter-chain coupling}}. \qquad \text{(S16)}
\end{aligned}$$

Here, the spin Hamiltonian is transformed into two Kitaev chains with distinct coupling strengths J_e and J_o, where onsite and inter-chain couplings are present with strengths h_x, V_{xx}, respectively. To see this result in the Majorana picture, we further transform each $c_i^\dagger, c_i$ into Majorana fermionic operators α_i,β_i by:

$$c_i^\dagger = \frac{\alpha_i - i\beta_i}{2}, \quad c_i = \frac{\alpha_i + i\beta_i}{2}. \qquad \text{(S17)}$$

Then, the fermionic Hamiltonian in Eq. (S16) is mapped into:

$$H_\mathrm{mf} = -i\sum_{i=1}^{N/2-1}(J_\mathrm{e}\beta_{2i-1}\alpha_{2i+1} + h_x\alpha_{2i-1}\beta_{2i-1}) - ih_x\alpha_{N-1}^\dagger\beta_{N-1} - i\sum_{i=1}^{N/2-1}(J_\mathrm{o}\beta_{2i}\alpha_{2i+2} + h_x\alpha_{2i}\beta_{2i}) - ih_x\alpha_N^\dagger\beta_N + V_{xx}\sum_{i=1}^{N-1}\alpha_i\beta_i\alpha_{i+1}\beta_{i+1}. \qquad \text{(S18)}$$

In summary, the total transformation is given by

$$\sigma_i^x = -i\alpha_i\beta_i, \quad \sigma_i^z = -\left[\prod_{j=1}^{i-1}(-i\alpha_j\beta_j)\right]\alpha_i. \qquad \text{(S19)}$$

We illustrate how each term in the spin Hamiltonian H mapped into Majorana fermionic operators in Fig. S4**a**, and the two coupled Kitaev chains after the transformation is shown in Fig. S4**b**.

A few remarks are in order. First, the edge operators originally defined in the spin Hamiltonian are now mapped into the following form:

$$\tilde{Z}_\mathrm{L} = -\alpha_1, \quad \tilde{X}_\mathrm{L} = -\alpha_2, \quad \tilde{Z}_\mathrm{R} = -i\left[\prod_{j=1}^{N}(-i\alpha_j\beta_j)\right]\beta_N, \quad \tilde{X}_\mathrm{R} = -i\left[\prod_{j=1}^{N}(-i\alpha_j\beta_j)\right]\beta_{N-1}. \qquad \text{(S20)}$$

While the left edge operators are directly represented by Majorana edge modes α_1, α_2 in each Kitaev chain, there is an additional term $\prod_{j=1}^{N}(-i\alpha_j\beta_j)$ at right edges. Notably, this is the generator for the total $\mathbb{Z}_2$ symmetry:

$$G = \prod_{j=1}^{N}(-i\alpha_j\beta_j) = G_\mathrm{e}G_\mathrm{o}, \quad G_\mathrm{e} = \prod_{i=1}^{N/2}(-i\alpha_{2i}\beta_{2i}), \quad G_\mathrm{o} = \prod_{i=1}^{N/2}(-i\alpha_{2i-1}\beta_{2i-1}). \qquad \text{(S21)}$$

As the system preserves G_e and G_o, it also preserves G. Therefore, the right edge operator $\tilde{Z}_\mathrm{R}$, $\tilde{X}_\mathrm{R}$ are solely determined by the state of β_N, β_{N-1} during the evolution.

Second, the single-body and two-body terms in H_1 are mapped into onsite and inter-chain coupling terms. Given that h_x and V_{xx} are small, the system keeps in the topological phase with $\alpha_1, \alpha_2, \beta_{2N-1}, \beta_{2N}$ nearly unpaired. In addition, the first-order

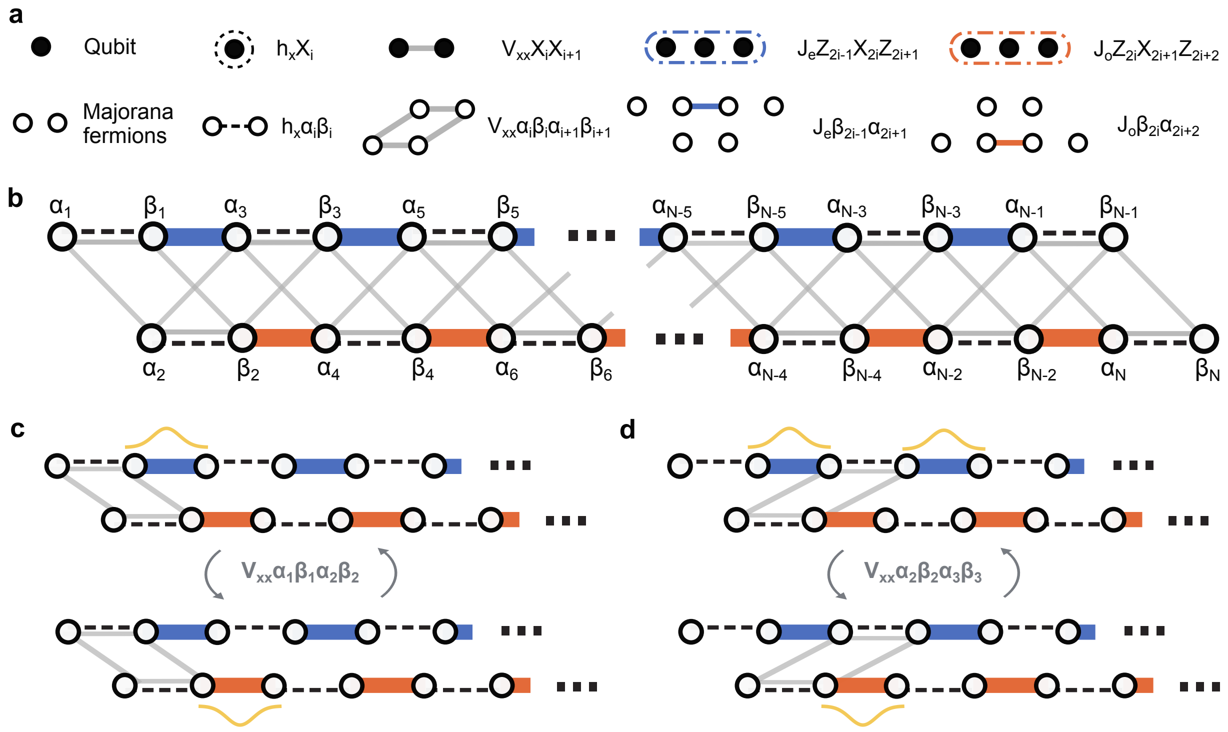

Fig. S4. **The SPT spin chain in the Majorana fermion picture.** **a,** The correspondence between each term in spin Hamiltonian H and Majorana operators α, β. Each qubit is non-locally transformed into two Majorana fermions. Single-body σ_i^x and two-body $\sigma_i^x\sigma_{i+1}^x$ terms are transformed into onsite and inter-chain couplings involving two and four Majoranas, respectively. The three-body stabilizers on even and odd sites are mapped into couplings between two Majoranas on different sites. **b,** The SPT spin chain is transformed into two Kitaev chains with inter-site coupling strengths J_e (upper chain) and J_o (lower chain). **c-d,** The first-order resonances between two Kitaev chains for (**c**) $J_\mathrm{o} = J_\mathrm{e}$ and (**d**) $J_\mathrm{o} = 2J_\mathrm{e}$.

resonance we discussed above is better understood in the Majorana fermion picture. The $\sigma_1^x\sigma_2^x$ term now becomes $\alpha_1\beta_1\alpha_2\beta_2$, pairing α_1, α_2 and exchanging the occupation between $J_\mathrm{e}\beta_1\alpha_3$ and $J_\mathrm{o}\beta_2\alpha_4$ (Fig. S4**c**). The $\sigma_2^x\sigma_3^x$ term reads $\alpha_2\beta_2\alpha_3\beta_3$, which involves α_2 and transfers both occupied $J_\mathrm{e}\beta_1\alpha_3$ and $J_\mathrm{e}\beta_3\alpha_5$ into $J_\mathrm{o}\beta_2\alpha_4$ (Fig. S4**d**). These readily give the first-order resonant conditions $J_\mathrm{o} = J_\mathrm{e}$ and $J_\mathrm{o} = 2J_\mathrm{e}$.

Third, in the Majorana picture, the U(1)×U(1) symmetry now represents the conservation laws for occupation numbers of inter-site couplings within each Kitaev chain, i.e. $\sum_{i=1}^{N-1}\beta_{2i-1}\alpha_{2i+1}$ and $\sum_{i=1}^{N-1}\beta_{2i}\alpha_{2i+2}$. As a result, the $\mathbb{Z}_2$ charges in the bulk of each chain are also conserved:

$$\mathcal{F}_\mathrm{o} = \prod_{i=1}^{N/2-1}\beta_{2i-1}\alpha_{2i+1} = \beta_1\left(\prod_{i=2}^{N/2-1}\alpha_{2i-1}\beta_{2i-1}\right)\alpha_{N-1}, \quad \mathcal{F}_\mathrm{e} = \prod_{i=1}^{N/2-1}\beta_{2i}\alpha_{2i+2} = \beta_2\left(\prod_{i=2}^{N/2-1}\alpha_{2i}\beta_{2i}\right)\alpha_N. \tag{S22}$$

As the U(1)×U(1) symmetry emerges in the dimerized and off-resonant region, the system commutes with all of the symmetries G_e, G_o, $\mathcal{F}_\mathrm{e}$, and $\mathcal{F}_\mathrm{o}$. This gives the conserved $\mathcal{F}_\mathrm{o}G_\mathrm{o} = (-i)^{N/2}\alpha_1\beta_{2N-1}$ and $\mathcal{F}_\mathrm{e}G_\mathrm{e} = (-i)^{N/2}\alpha_2\beta_{2N}$. Since α_1 (α_2) and β_{2N-1} (β_{2N}) are at two ends of the chain separated by N sites, and perturbations are local, we conclude that each of α_1, α_2, β_{2N-1} and β_{2N} is conserved.

G. Energy spectroscopy in an integrable chain

Here, we briefly explain how we measure the single-particle spectrum. First, we note that the Jordan-Wigner transformation in Eq. (S14) can also be applied to the quantum circuits in our experiments. In the limit with $V_{xx} = 0$, the unitary after transformation reads,

$$U_\mathrm{f} = \left[\prod_{i=1}^{N/2-1} e^{i\delta t J_\mathrm{e}(c_{2i-1}^\dagger - c_{2i-1})(c_{2i+1}^\dagger + c_{2i+1})}\prod_{i=1}^{N/2} e^{-i\delta t h_x(1-2c_{2i-1}^\dagger c_{2i-1})}\right]\left[\prod_{i=1}^{N/2-1} e^{i\delta t J_\mathrm{o}(c_{2i}^\dagger - c_{2i})(c_{2i+2}^\dagger + c_{2i+2})}\prod_{i=1}^{N/2} e^{-i\delta t h_x(1-2c_{2i}^\dagger c_{2i})}\right] = U_{\mathrm{K,e}}U_{\mathrm{K,o}}, \tag{S23}$$

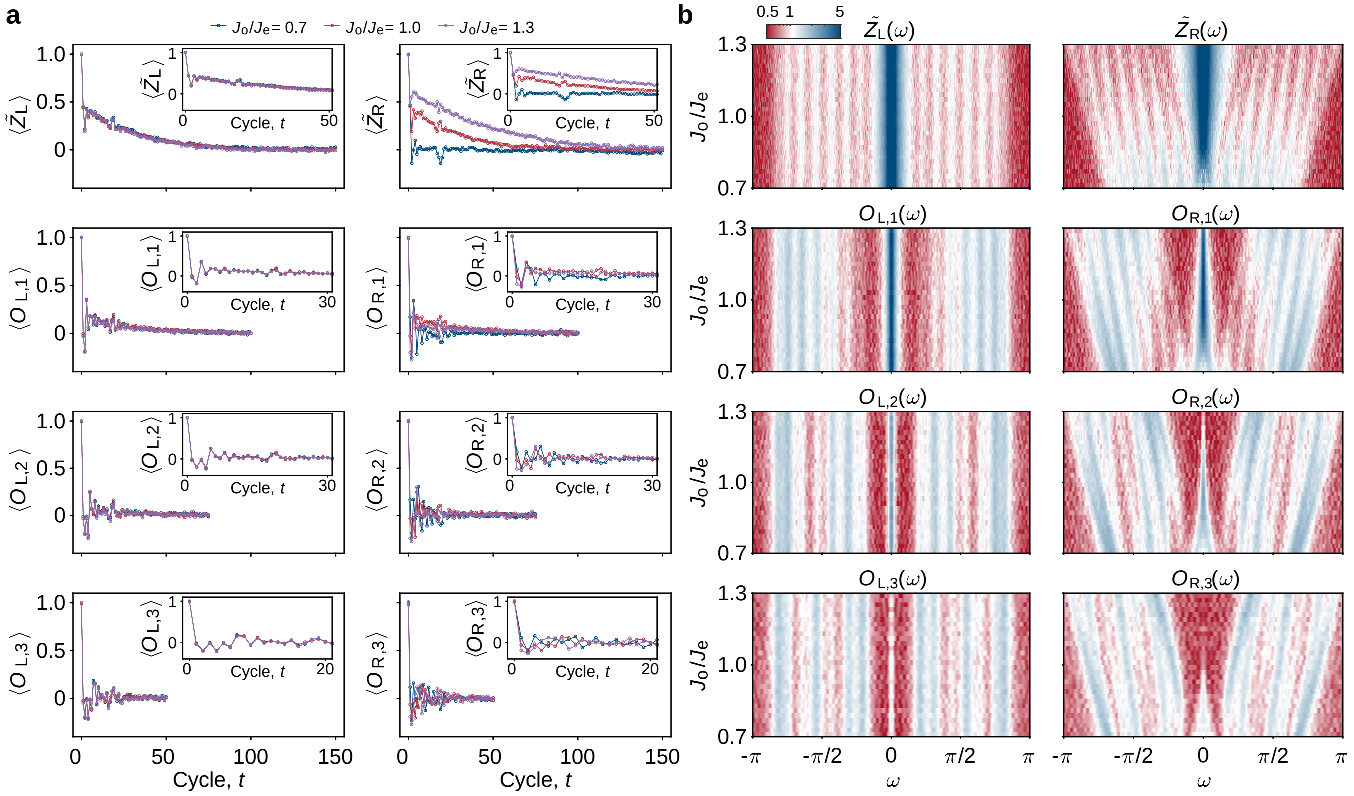

Fig. S5. **Energy spectroscopy of the** 16**-qubit system**. **a**, Time-domain signals of $\langle O_{L,i}(t)\rangle$ (left panel) and $\langle O_{R,i}(t)\rangle$ (right panel) for the integrable chain ($V_{xx} = 0$). We fix $J_e = \pi/2$, $h_x = 7\pi/20$ and vary J_o/J_e by tuning J_o. In each case, the initial states are product states with the expectation value of the corresponding operator is 1 at $t = 0$. **b**, Frequency-domain signals of $O_{L,i}(\omega)$ (left panel) and $O_{R,i}(\omega)$ (right panel).

with mutually commuting $U_{K,e}$ and $U_{K,o}$. $U_{K,e}$, $U_{K,o}$, which are called kicked Kitaev models, are widely studied in theory [S15–S18]. This model is exactly solvable with Bogoliubov eigenmodes $\nu^\dagger, \nu$. For $U_{K,e}$ and $U_{K,o}$, we have

$$U^\dagger_{K,e}\nu_{2i-1}U_{K,e} = e^{-i\epsilon_{2i-1}}\nu_{2i-1}, \quad \nu_{2i-1} = \sum_{j=1}^{N/2} u_{2i-1,2j-1}c^\dagger_{2j-1} + v_{2i-1,2j-1}c_{2j-1}. \tag{S24}$$

$$U^\dagger_{K,o}\nu_{2i}U_{K,o} = e^{-i\epsilon_{2i}}\nu_{2i}, \qquad \nu_{2i} = \sum_{j=1}^{N/2} u_{2i,2j}c^\dagger_{2j} + v_{2i,2j}c_{2j}. \tag{S25}$$

Notably, as two kicked Kitaev chains are decoupled, the Bogoliubov eigenmodes are constructed from the fermionic operators within each chain, leading to

$$[U_{K,e}, \nu_{2i}] = 0, \quad [U_{K,o}, \nu_{2i-1}] = 0. \tag{S26}$$

Now consider the dynamics of the following set of operators:

$$O_{L,i} = \left(\prod_{k=1}^{2i}\sigma^x_k\right)\sigma^z_{2i+1}, \quad O_{R,i} = \left(\prod_{k=1}^{2i}\sigma^x_{2N+1-k}\right)\sigma^z_{2(N-i)}. \tag{S27}$$

For $i = 0$, they are exactly the edge operators $\tilde{Z}_L, \tilde{Z}_R$ and for $i > 0$, they are the bulk terms in the PSZMs when H_1 only contains single-qubit σ^x_i perturbations. Under the Jordan-Wigner transformation, $O_{L,i}, O_{R,i}$ are mapped to the fermionic operators in the upper and lower Kitaev chains, respectively:

$$O_{L,i} = -(c_{2i+1} + c^\dagger_{2i+1}) = \sum_{j=1}^{N/2} l_{i,j}\nu_{2j-1} + l^*_{i,j}\nu^\dagger_{2j-1}, \quad O_{R,i} = -G(c_{2(N-i)} + c^\dagger_{2(N-i)}) = G\sum_{j=1}^{N/2} r_{i,j}\nu_{2j} + r^*_{i,j}\nu^\dagger_{2j}, \tag{S28}$$

where $G = \prod_i \sigma_i^x$ is preserved as the system preserves the $\mathbb{Z}_2 \times \mathbb{Z}_2$ symmetry. As the decomposition of $O_{\mathrm{L},i}$ ($O_{\mathrm{R},i}$) only involves the Bogoliubov eigenmodes within each Kitaev chain, their spectra can be reconstructed separately from the operator dynamics. For an arbitrary initial state $|\psi_0\rangle$, the dynamics read

$$\langle\psi_0|O_{\mathrm{L},i}(t)|\psi_0\rangle = \sum_{j=1}^{N/2} \left\langle \psi_0 \left| (U_{\mathrm{K,o}}^\dagger U_{\mathrm{K,e}}^\dagger)^t (l_{i,j}\nu_{2j-1} + l_{i,j}^* \nu_{2j-1}^\dagger)(U_{\mathrm{K,e}} U_{\mathrm{K,o}})^t \right| \psi_0 \right\rangle = \sum_{j=1}^{N/2} l_{i,j} \langle\psi_0|\nu_{2j-1}|\psi_0\rangle\, e^{-it\epsilon_{2j-1}} + \text{h.c.}, \tag{S29}$$

$$\langle\psi_0|O_{\mathrm{R},i}(t)|\psi_0\rangle = \sum_{j=1}^{N/2} \left\langle \psi_0 \left| (U_{\mathrm{K,o}}^\dagger U_{\mathrm{K,e}}^\dagger)^t G (r_{i,j}\nu_{2j} + r_{i,j}^* \nu_{2j}^\dagger)(U_{\mathrm{K,e}} U_{\mathrm{K,o}})^t \right| \psi_0 \right\rangle = \sum_{j=1}^{N/2} r_{i,j} \langle\psi_0|G\nu_{2j}|\psi_0\rangle\, e^{-it\epsilon_{2j}} + \text{h.c.}, \tag{S30}$$

where h.c. denotes Hermitian conjugate terms. After Fourier transformations, the spectrum $\{\epsilon_{2j-1}\}$ $[\{\epsilon_{2j}\}]$ of the kicked Kitaev chain with inter-site coupling strength J_e $[J_\mathrm{o}]$ is revealed by $O_{\mathrm{L},i}(\omega)$ $[O_{\mathrm{R},i}(\omega)]$. The Fourier amplitude of each ϵ_i is determined by both the overlaps between Bogoliubov eigenmodes ν_i and original fermionic operators $c_i, c_i^\dagger$, and the overlaps between ν_i and the initial state $|\psi_0\rangle$. Therefore, we can select different operators $O_{\mathrm{L},i}, O_{\mathrm{R},i}$, or prepare different initial states to enhance the detection of specific eigenmodes, enabling us to perform spectroscopy on larger systems.

In our experiment, we fix h_x, J_e with varying J_o. For the 16-qubit chain, we measure $O_{\mathrm{L},i}, O_{\mathrm{R},i}$ up to $i = 3$, with initial states being product states such that $\langle O_{\mathrm{L},i}(0)\rangle = \langle O_{\mathrm{R},i}(0)\rangle = 1$. Their time dynamics and Fourier transforms are presented in Fig. S5. Notably, only the dynamics of $O_{\mathrm{R},i}$ change with varying J_o, and different $O_i(\omega)$ more effectively characterize different bulk eigenmodes. The spectra displayed in the main text are obtained from averaging $\tilde{Z}(\omega)$ and $O_i(\omega)$.

H. Noiseless simulation via matrix product states

For our 100-qubit system, it is quite challenging to carry out numerical simulations with noise to validate the experimental results. Nevertheless, it could still be feasible to compare the error-mitigated results of excitation numbers, which are calculated from $\langle \overline{K_i}\rangle = \langle\Psi_\mathrm{e}|K_i(t)|\Psi_\mathrm{e}\rangle \,/\, \langle\Psi_0|K_i(t)|\Psi_0\rangle$, with the noiseless simulation results. To this end, we use the matrix product state (MPS) [S19, S20] to simulate the system dynamics without noise. We first use MPS to calculate the dynamics of a small system with $N = 12$ qubits, which is accessible with exact diagonalization (ED) and yields a comparison for benchmarks. As shown in Fig. S6a, MPS results (black lines) and ED results (green dots) agree precisely with each other. For the homogeneous case ($J_\mathrm{o}/J_\mathrm{e} = 1$, left panel in Fig. S6a), we observe a rapid relaxation of the edge mode $\tilde{Z}_\mathrm{L}$, as well as excitation numbers on odd

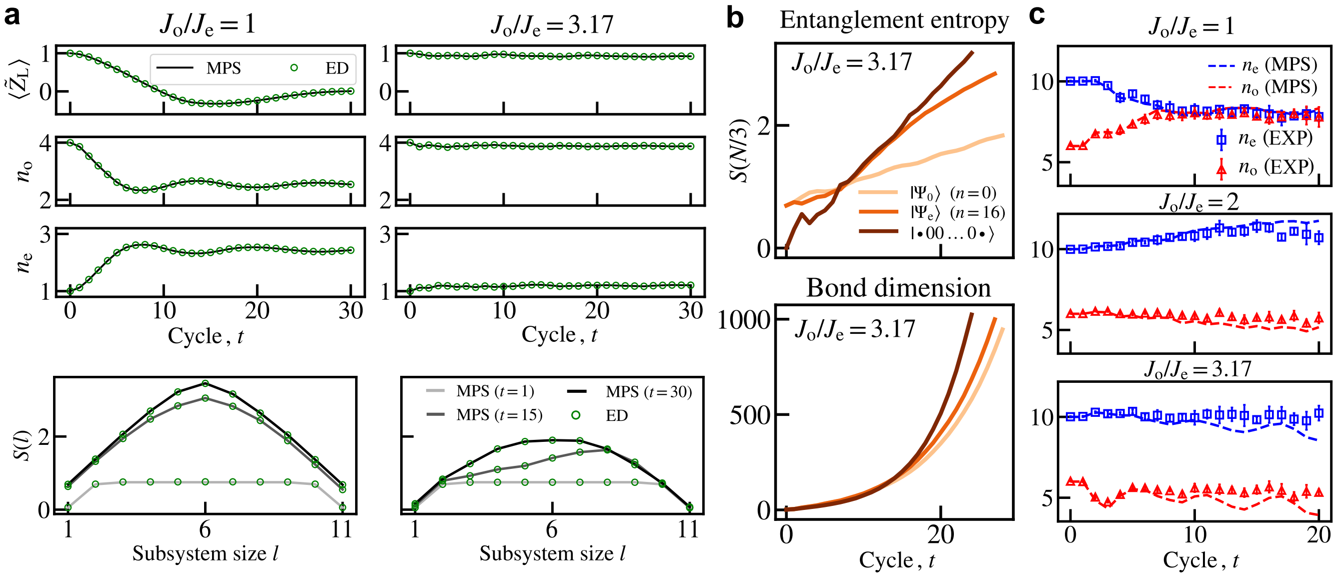

Fig. S6. **Matrix product state simulation results.** **a**, Comparison between the results obtained from matrix product state (MPS) and exact diagonalization (ED) for a small system with $N = 12$ qubits. The initial state is a cluster state with 5 excitations. **b**, The growth of entanglement entropy and bond dimensions for three different initial states in our $N = 100$ qubit experiment. **c**, Comparison between the MPS results and experimental data for the emergent U(1)×U(1) symmetry. In both cases, the excitation numbers are obtained from the normalized expectation values of stabilizers $\langle \overline{K_i}\rangle = \langle\Psi_\mathrm{e}|K_i(t)|\Psi_\mathrm{e}\rangle \,/\, \langle\Psi_0|K_i(t)|\Psi_0\rangle$. The small deviations between the MPS and experimental data after around 15 cycles are due to accumulations of experimental imperfections. In all cases, the truncation error cutoff is set to be 10^{-6}.

sites, n_o, and even sites, n_e. As expected, the entanglement entropy S also increases rapidly and almost saturates at $t = 30$ cycles. For the dimerized case ($J_o/J_e = 3.17$, right panel in Fig. S6a), while $\tilde{Z}_L$, n_o, and n_e are approximately conserved, S still grows considerably during the evolution. This growth occurs because excitations can propagate freely among the sites with the same parity, even though the dimerization suppresses edge-bulk interactions and excitation exchanges between sites with different parity (as shown in Fig. 3 of the main text).

We then use MPS to simulate 100-qubit system. In Fig. S6b, we observe a nearly linear growth of entanglement entropy S for the dimerized case. This is in sharp contrast to the case of many-body localization, where propagation of excitations is forbidden, and the entanglement entropy grows logarithmically with time. In addition, the growth rate of S increases as the number of excitations in the initial state rises, which poses challenges for simulating the long-time dynamics of our system. Indeed, we find that for fixed truncation error, 10^{-6}, the bond dimensions quickly grow up to 1024 and become intractable when $t \gtrsim 30$. This aligns with the recent experimental progress [S21], which demonstrated that accurately simulating prethermal states using current classical methods is quite challenging. Nevertheless, we can still use the MPS results to verify our experimental data for short timescales. In Fig. S6c, we compare the time dynamics of n_e, n_o between MPS results (dashed lines) and experimental data (markers). These results agree well with each other, further confirming the presence of U(1)×U(1) symmetry and the performance of our device.

2. EXPERIMENTAL INFORMATION

A. Device performance

As shown in Fig. 1**a** of the main text, we construct a one-dimensional chain with 100 qubits on our 125-qubit quantum processor to implement the theoretical model. The wiring information for our device and room-temperature control electronics are sketched in Fig. S7. Figure S8**a** displays the typical idle frequencies of the 100 qubits, where we apply single-qubit gates in our experiments. The measured energy relaxation time T_1 and spin-echo dephasing time T_2^{SE} at idle frequencies in Fig. S8**a** are listed in Fig. S9, whose median values are about 70.3 μs and 17.5 μs, respectively. Figure S8**b** shows the readout error for each qubit, which is defined as the average error for measuring $|0\rangle$ state and $|1\rangle$ state. They are measured by preparing the 100 qubits in random product states $\{|0\rangle, |1\rangle\}^{\otimes 100}$ and averaging the outcome for each qubit. We note that an extra microwave pulse that yields $|1\rangle \leftrightarrow |2\rangle$ transition is applied to each qubit before the readout microwave pulse to improve readout fidelity. The median value of readout errors is 0.88%.

B. Gate calibration

In our experiments, single-qubit gates are realized by 20-ns microwave pulses, which have Gaussian envelope modulated with the derivative reduction by adiabatic gate (DRAG) pulse. We compile consecutive single-qubit gates into a single-qubit rotation $U3(\theta, \varphi, \lambda)$ with the following matrix form

$$U3(\theta,\varphi,\lambda) = e^{-i\frac{\varphi}{2}\sigma_z} e^{-i\frac{\theta}{2}\sigma_y} e^{-i\frac{\lambda}{2}\sigma_z} = \begin{pmatrix} \cos\frac{\theta}{2} & -e^{i\lambda}\sin\frac{\theta}{2} \\ e^{i\varphi}\sin\frac{\theta}{2} & e^{i(\varphi+\lambda)}\cos\frac{\theta}{2} \end{pmatrix} \quad \text{(up to global phase).} \tag{S31}$$

In practice, $U3(\theta, \varphi, \lambda)$ gate is realized by a virtual phase gate and a subsequent XY rotation. CPhase(ϕ) gates (ϕ is the conditional phase, $\phi \in \{\pi, -0.4\}$ in our experiments) are realized by tuning $|11\rangle$ and $|20\rangle$ states of the two qubits near resonance and switching on the coupling between them for a certain duration. Experimentally, we achieve a specific ϕ by tuning the Z-pulse amplitudes (amplitudes of the flux pulses input from fast Z-pulse lines in Fig. S7) of the qubits and coupler. The pulse durations for CZ and CPhase(-0.4) gates are 40 ns and 34 ns, respectively. As shown in Fig. S11, implementing a single Trotter step $U(\delta t)$ requires four layers of CZ gates, two layers of CPhase(-0.4) gates, and three layers of single-qubit gates, which in total corresponds to a sequence of 288-ns duration, placing high demands on gate fidelity.

For CPhase(ϕ) gates with ϕ close to π (e.g., $|\phi - \pi| < 2$ in our case), we usually calibrate Z-pulse amplitudes of qubits either by maximizing qubit entanglement [S22] or Floquet calibration [S23, S24]. The Z-pulse amplitude of coupler is determined by minimizing the leakage to $|2\rangle$ state. However, for the cases of $|\phi - \pi| \geq 2$, the low-leakage area becomes broad, making it hard to identify the right parameters [S25]. Here, we propose a sequence to characterize CPhase gates for an arbitrary conditional phase ϕ (Fig. S10), which we use to calibrate the Z-pulse amplitudes of qubits. Specifically, for CPhase(-0.4) gates, we start from the control parameters of CZ gates, and then use Floquet calibration to initialize the Z-pulse amplitudes of qubits so that the conditional phase is around 4.5. Further, we utilize the sequence described in Fig. S10 to calibrate the Z-pulse amplitude of the coupler to achieve the target conditional phase of -0.4.

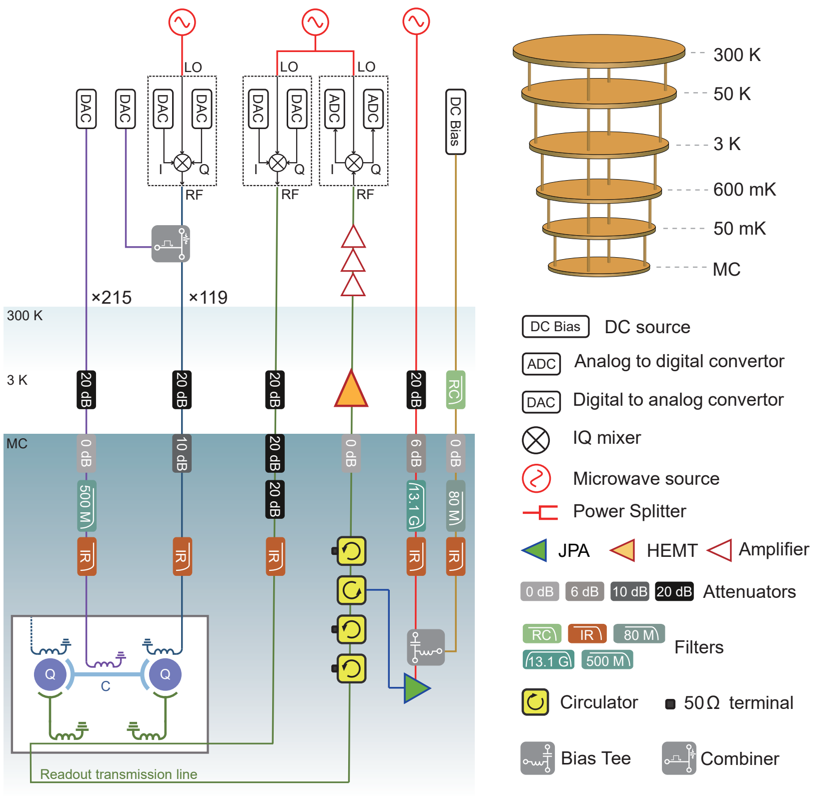

Fig. S7. **Experimental setup.** Quantum processor, denoted by the white box at the bottom left corner, is mounted on the mixing chamber plate (MC) of the dilution refrigerator, whose base temperature is around 20 mK. Fast Z-pulse lines (purple), microwave-drive lines (blue), and readout lines (green) connect room-temperature electronics to the processor for control and measurement. Details of the microwave components are provided in the legend on the right.

C. Experimental circuits

In this section, we illustrate details about the quantum circuits used in our experiments. Figure S11**a** and **b** show the quantum circuits for measuring $\tilde{Z}$ and $\tilde{X}$ operators in Fig. 2 and Fig. 3 of the main text with $t = 1$ (circuits with $t > 1$ are constructed by repeating the Trotter step circuit). In our experiments, these circuits will be further compiled to reduce the circuit depth. For example, the excitation gate $X(\pi)$ acting on Q_i in Fig. S11**a** is compiled into three gates $\{Z(\pi), X(\pi), Z(\pi)\}$ acting on $\{Q_{i-1}, Q_i, Q_{i+1}\}$ before the first layer of CZ gates. Then, these gates are merged into the Hadamard gate layer. Further, the CZ layers surrounded by the orange dashed frame in Fig. S11**a** can be eliminated because the two CZ gates on edges are redundant (edge qubits are in $|0\rangle$ state) and the rest CZ gates in the bulk can cancel out with each other. This similar elimination is also applied to the circuit in Fig. S11**b** as well. Note that the two-qubit gates in the Trotter steps of $t > 1$ cannot be eliminated.

After compilation, we utilize the Pauli twirling technique to suppress the damaging coherent noise, which is realized by inserting random single-qubit gates before and after each two adjacent CZ layers. To protect the edge qubits from dephasing noise, we also impose an extra restriction: A π-rotation is always embedded in the single-qubit layer that is sandwiched by the four CZ layers for the two edge qubits.

The echo evolution $U_{\text{echo}}(t) = (U^\dagger)^t U^t$ in the main text consists of t steps of forward time evolution U^t and the followed t steps of backward time evolution $(U^\dagger)^t$. Thus, the decay of echo evolution characterizes the accumulated circuit errors after initial state preparation. In our experiments, the circuit of U^t is defined as the circuit after the green dashed line in Fig. S11, with Pauli twirling gates also integrated into the backward time evolution.

D. Mitigation of leakage error

The experimental circuit for measuring the energy spectrum in Fig. 4 of the main text contains up to 150 Trotter steps with 600 layers of two-qubit gates. Such a long sequence makes the leakage to $|2\rangle$ state non-negligible, which systematically causes a slightly higher probability of qubit in $|1\rangle$ state. To suppress this effect, we also measure the $|2\rangle$ state probability in the many-body

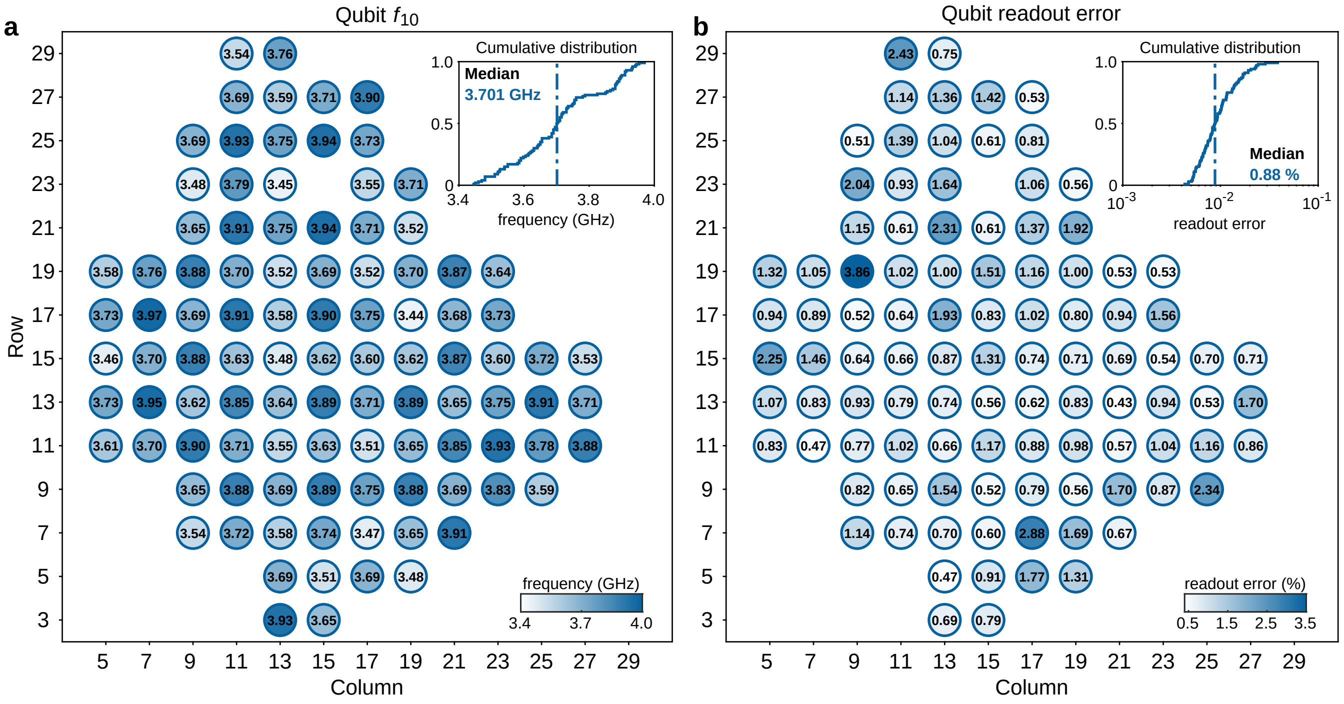

Fig. S8. **Qubit idle frequency and readout error.** **a**, Idle frequencies of the 100 qubits used in our experiments. Inset shows the cumulative distribution, with the dashed line indicating the median value. **b**, Qubit readout errors measured at idle frequencies in **a**. The data for each qubit is the average of the errors when qubit in state $|0\rangle$ and $|1\rangle$.

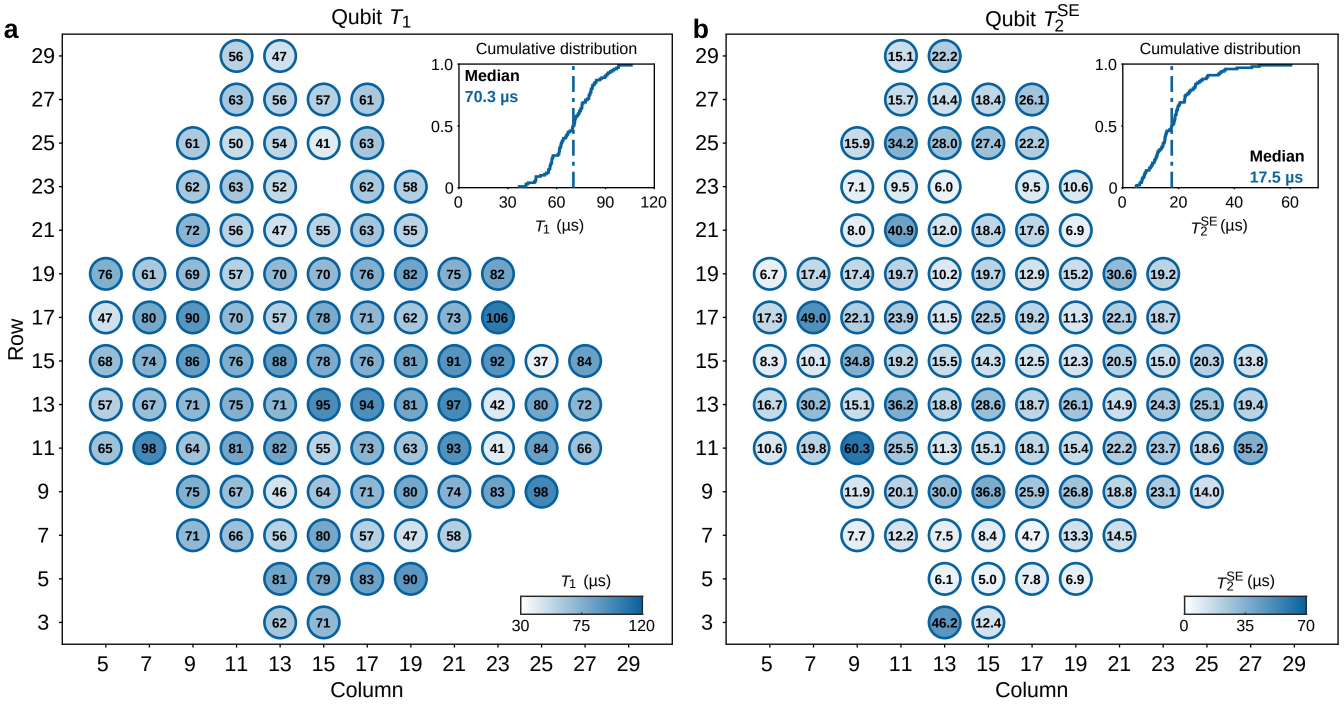

Fig. S9. **Qubit coherence time.** **a**, Energy relaxation time T_1 of the 100 qubits measured at idle frequencies, with a median value of 70.3 μs. **b**, Spin-echo dephasing time measured at idle frequencies, with a median value of 17.5 μs.

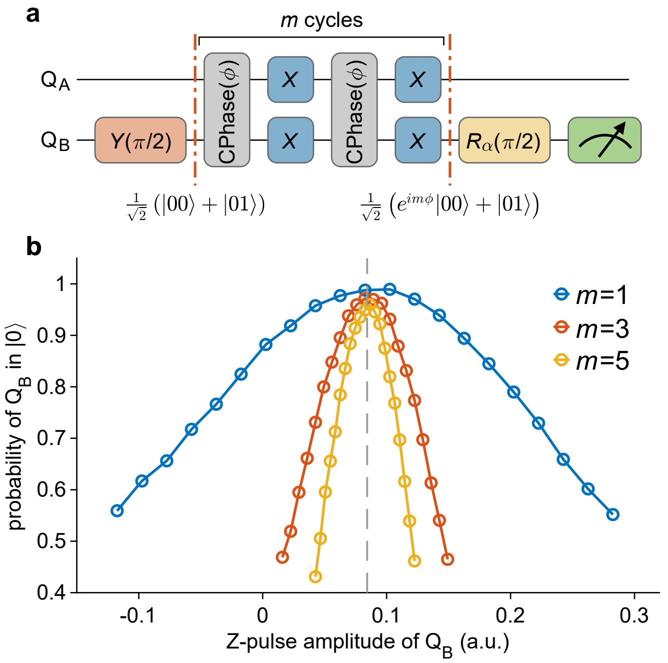

Fig. S10. **CPhase gate calibration.** **a**, Quantum circuit for calibrating CPhase(ϕ) gate. The circuit begins with a $Y(\pi/2)$ gate acting on Q_B, which prepares a superposition state $(|00\rangle + |01\rangle) / \sqrt{2}$. Then, m cycles of the interleaved circuit (including two layers of CPhase(ϕ) gates and two layers of single-qubit X gates) are applied, with each cycle accumulating a phase of ϕ on $|00\rangle$ state. Finally, an $R_\alpha(\pi/2)$ gate (a single-qubit rotation around the α axis of Bloch sphere, where α refers to an equatorial rotation axis that has an angle α with respect to the x axis) is applied to Q_B before readout. The measured probability of Q_B in $|0\rangle$ state is given by $[1 + \cos(\pi/2 + \alpha + m\phi)]/2$. In the ideal case without errors, by choosing $\alpha = -m\phi - \pi/2$, we should expect Q_B to be in $|0\rangle$ state with probability of 1. **b**, Measured probability of Q_B in $|0\rangle$ state as a function of the Z-pulse amplitude of Q_B. Gray dashed line indicates the calibrated Z-pulse amplitude for Q_B.

spectroscopy experiment and correct the experimental data. Taking one-body operators $\tilde{Z}_L$, $\tilde{Z}_R$ as an example, the procedure is as following:

- First, we run the experimental circuits and measure the probability of the qubit $\vec{P}_{\text{exp}} = \left(p_{0,\text{exp}}, p_{1,\text{exp}}, p_{2,\text{exp}}\right)^{\text{T}}$, where $p_{\alpha,\text{exp}}$ is the measured probability of qubit in $|\alpha\rangle$ state ($\alpha \in \{0, 1, 2\}$).

- Then, we use three-level readout correction matrix C to mitigate readout errors, which satisfies $C\vec{P}_{\text{ideal}} = \vec{P}_{\text{exp}}$ with the definition as below

$$C = \begin{pmatrix} 1 - \epsilon_{0\to1} - \epsilon_{0\to2} & \epsilon_{1\to0} & \epsilon_{2\to0} \\ \epsilon_{0\to1} & 1 - \epsilon_{1\to0} - \epsilon_{1\to2} & \epsilon_{2\to1} \\ \epsilon_{0\to2} & \epsilon_{1\to2} & 1 - \epsilon_{2\to0} - \epsilon_{2\to1} \end{pmatrix}, \tag{S32}$$

  where $\epsilon_{i\to j}$ refers to the measured probability of a qubit in $|j\rangle$ when it is prepared in $|i\rangle$ state. The matrix elements of C are benchmarked with a separate experiment. Thus, the estimated $\vec{P}_{\text{ideal}}$ after readout correction is given by $\vec{P}_{\text{corr}} = C_{\text{exp}}^{-1}\vec{P}_{\text{exp}}$, where C_{exp} is the experimentally measured readout correction matrix.

- To eliminate the state leakage error, we discard the probability in $|2\rangle$ state and normalize the measured probability in the computational space with the following equation

$$p_{0,\text{norm}} = \frac{p_{0,\text{corr}}}{p_{0,\text{corr}} + p_{1,\text{corr}}},\ p_{1,\text{norm}} = \frac{p_{1,\text{corr}}}{p_{0,\text{corr}} + p_{1,\text{corr}}}. \tag{S33}$$

- Finally, we calculate the expectation value $\langle\tilde{Z}\rangle = p_{0,\text{norm}} - p_{1,\text{norm}}$.

For many-body operators $O_{\text{L},i}$, $O_{\text{R},i}$ ($i > 0$), we follow the similar procedure described above. The difference is that the readout correction matrix has a dimension of $3^l \times 3^l$ for l-body operators, which is the tensor product of individual correction matrices of the l qubits involved.

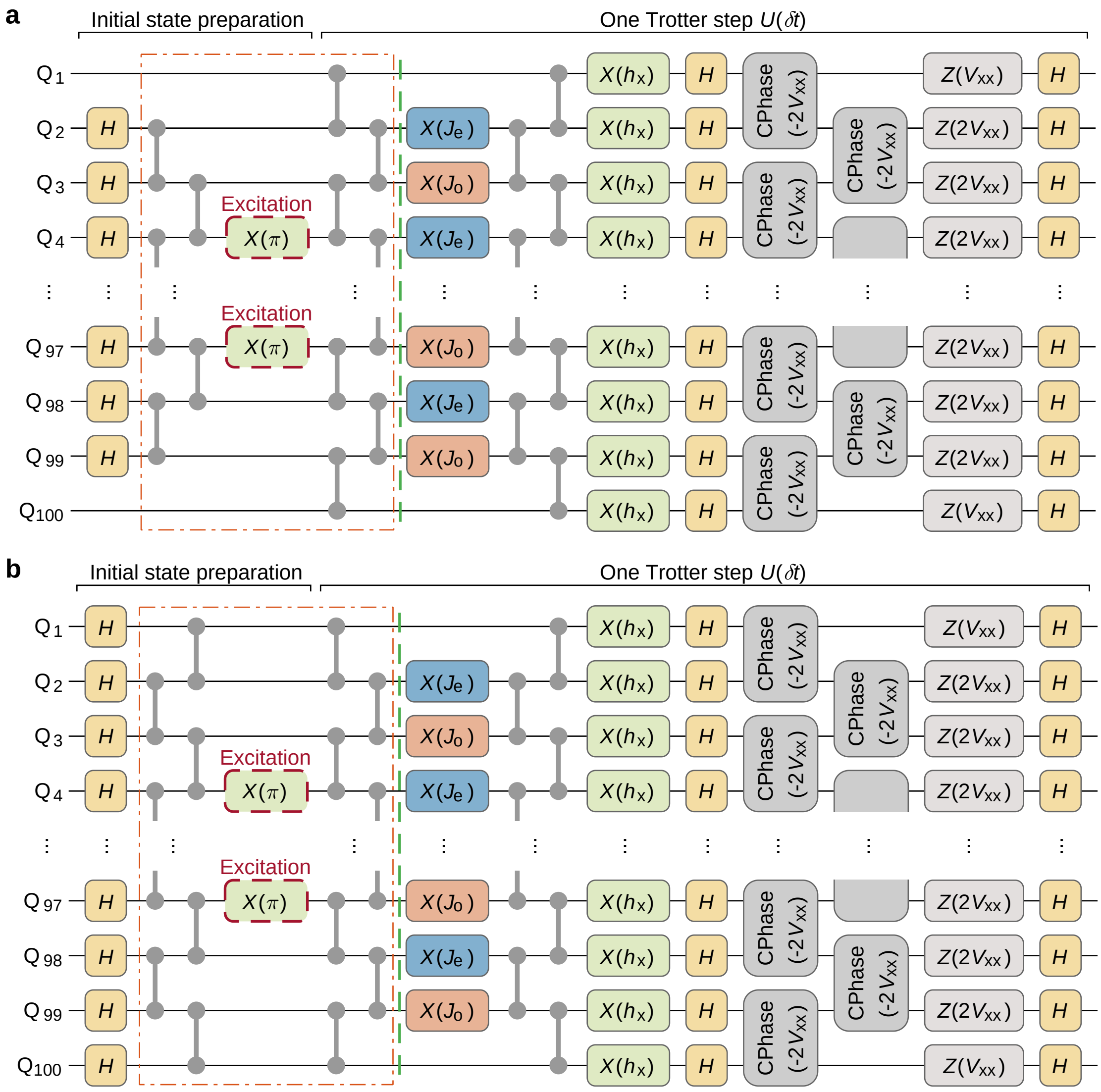

Fig. S11. **Experimental circuits.** **a**, Quantum circuit for measuring $\tilde{Z}$ operator in Fig. 2 of the main text when $t = 1$. The CZ gates in the orange dashed frame are canceled out after compilation in our experiments. The circuit on the right of the green dashed line corresponds to the unitary U^t as the building block of the echo circuit $U_{\text{echo}}(t) = (U^\dagger)^t U^t$. **b**, Quantum circuit for measuring $\tilde{X}$ operator in Fig. 2 and Fig. 3 of the main text when $t = 1$.

E. Quantum state tomography

In this section, we provide details about how we obtain the density matrix in the main text. To investigate the dynamics of logical state fidelity (Fig. 5**a** of the main text), it is natural to perform two-qubit logical state tomography on the two edge states and reconstruct the full logical density matrix ρ_{logic}, then calculate the logical state fidelity $F\left(\rho_{\text{logic}}, \rho_{\text{ideal}}\right)$ (ρ_{ideal} is the ideal density matrix of the logical state) by the following formula

$$\rho_{\text{logic}} = \frac{1}{4}\left(\sum_{\tilde{P}_{\text{L}},\tilde{P}_{\text{R}}\in\{I,\tilde{X},\tilde{Y},\tilde{Z}\}} \langle \tilde{P}_{\text{L}}\tilde{P}_{\text{R}}\rangle\, \tilde{P}_{\text{L}}\tilde{P}_{\text{R}}\right), \tag{S34}$$

$$F\left(\rho_{\text{logic}}, \rho_{\text{ideal}}\right) = \text{tr}\left(\sqrt{\sqrt{\rho_{\text{logic}}}\rho_{\text{ideal}}\sqrt{\rho_{\text{logic}}}}\right)^2, \tag{S35}$$

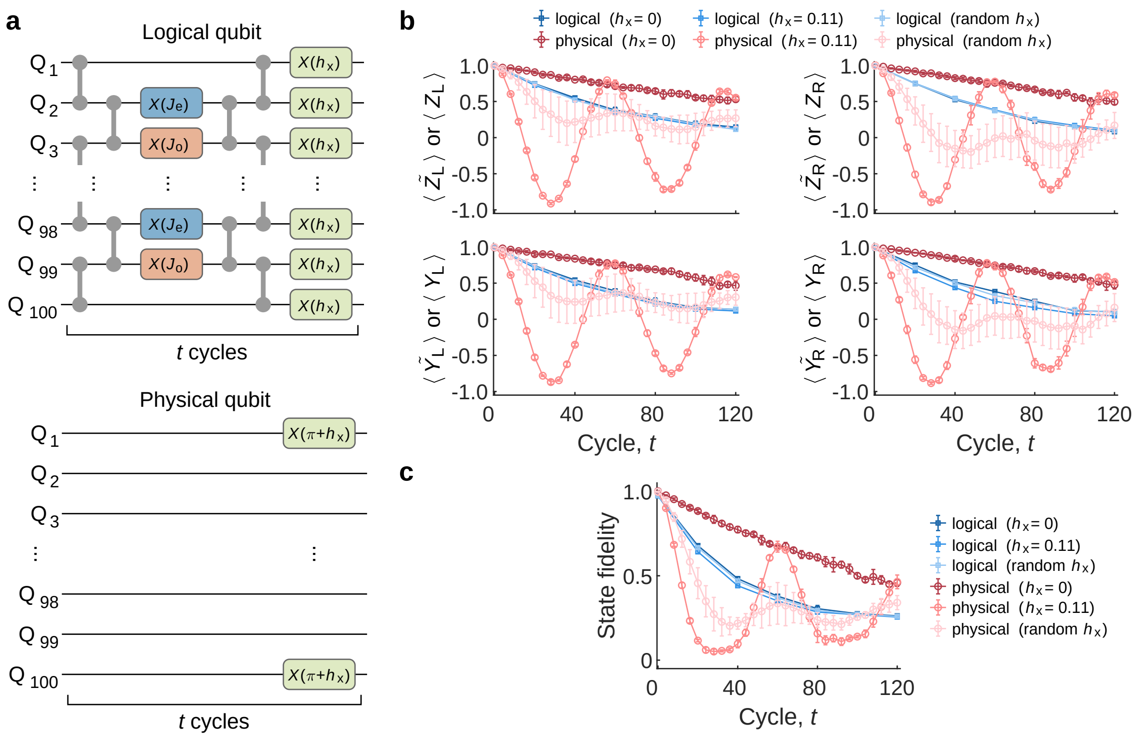

Fig. S12. **Comparison of noise resilience between logical and physical qubits.** **a**, Experimental circuits for time evolution of logical (top panel) and physical (bottom panel) qubits, with execution time per cycle equal to 200 ns for both cases. For logical qubits, we set $J_o = 3.17J_e = 3.17\pi/5$. The initial states of logical (physical) qubits are prepared in either $|\tilde{0}\rangle_L\,|\tilde{0}\rangle_R$ ($|00\rangle$) for measuring $\langle\tilde{Z}\rangle$ ($\langle Z\rangle$), $(|\tilde{0}\rangle_L + \mathrm{i}\,|\tilde{1}\rangle_L)\otimes(|\tilde{0}\rangle_R + \mathrm{i}\,|\tilde{1}\rangle_R)$ $[(|0\rangle + \mathrm{i}\,|1\rangle)\otimes(|0\rangle + \mathrm{i}\,|1\rangle)]$ for measuring $\langle\tilde{Y}\rangle$ $[\langle Y\rangle]$, or $|\tilde{0}\rangle_L\,|\tilde{0}\rangle_R + \mathrm{i}\,|\tilde{1}\rangle_L\,|\tilde{1}\rangle_R$ $(|00\rangle + \mathrm{i}\,|11\rangle)$ for Bell state fidelity. **b**, Measured dynamics of logical and physical operators with ($h_x = 0.11$, or random h_x uniformly chosen from $[-0.11, 0.11]$) and without ($h_x = 0$) perturbations. **c**, Fidelity dynamics of logical and physical Bell states. Error bars in **b** and **c** represent the standard deviation (for $h_x = 0$ and $h_x = 0.11$) or the standard error of the statistical mean (for random h_x) over five rounds of measurements, with each round taking 10,000 shots for logical qubits and 3,000 shots for physical qubits.

where $\tilde{X}, \tilde{Y}, \tilde{Z}$ are logical Pauli operators. To obtain the fidelity of logical Bell state $(|\tilde{0}\rangle_L\,|\tilde{0}\rangle_R + \mathrm{i}\,|\tilde{1}\rangle_L\,|\tilde{1}\rangle_R)$ in Fig. 5**a** of the main text, we simplify the measurement of $F\left(\rho_{\text{logic}}, \rho_{\text{ideal}}\right)$ by only probing three logical Pauli strings

$$F_{\text{Bell}} = \frac{1}{4}\left(1 + \langle\tilde{X}_L\tilde{Y}_R\rangle + \langle\tilde{Y}_L\tilde{X}_R\rangle + \langle\tilde{Z}_L\tilde{Z}_R\rangle\right). \tag{S36}$$

However, in Fig. 5**b** of the main text and Extended Data Fig. 8**b**, we measure all the logical Pauli operators in Eq. S34 to reconstruct all the elements of the full density matrices. In Extended Data Fig. 8**c**, we perform full quantum state tomography of the four physical qubits $Q_1, Q_2, Q_{99}, Q_{100}$ at edges and reconstruct its density matrix with a similar method of Eq. S34. The reconstructed full density matrices are further validated in the constraints of Hermitian, unit trace, and positive semi-definite with the method described in Ref. [S26].

F. Comparison of noise resilience between edge modes and physical qubits

In theory, the edge modes of the SPT Hamiltonian are robust against local, symmetry-preserving perturbations (e.g., the $h_x\sigma_i^x$ and $V_{xx}\sigma_i^x\sigma_{i+1}^x$ terms considered in H_1). While these perturbations can induce hybridization of edge modes, the resulting lifetime t_N is predicted to be finite but exhibits exponential scaling with the system size N. In contrast, without applying elaborately designed dynamical-decoupling sequences, the unprotected physical qubits are vulnerable: a constant local single-body σ_i^x perturbation directly induces oscillations, leading to an immediate loss of encoded information. Similarly, unexpected two-body $\sigma_i^x\sigma_{i+1}^x$ couplings between physical qubits could also cause rapid decoherence.

Here, we give an experimental demonstration of this noise resilience of edge modes. The circuits we applied are depicted in Fig. S12**a**, where edge modes evolve under the SPT Hamiltonian H_0 and the physical qubits are subjected to Rabi π pulses. In each scenario, we introduced a layer of R_x rotation gates to mimic symmetry-preserving noise with tunable strength h_x.

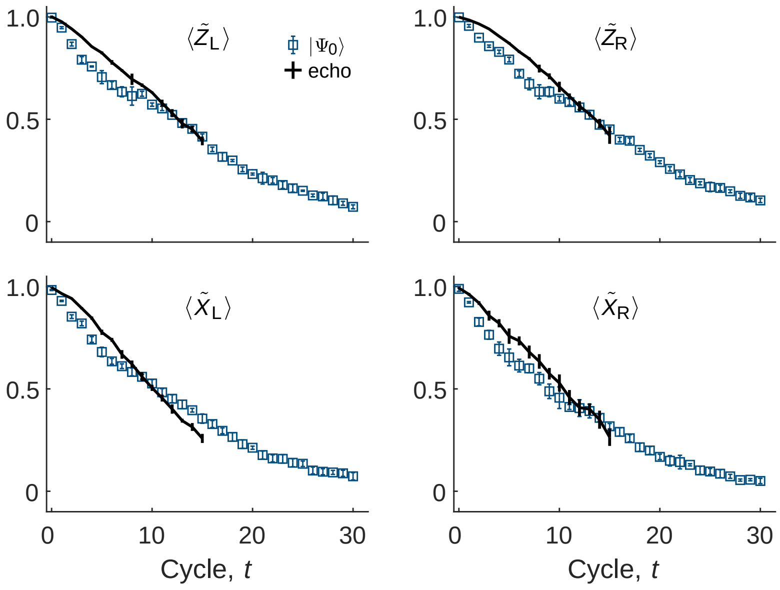

Fig. S13. Initial experimental results of edge operators for the case of $\{|\Psi_0\rangle\}$ and echo experiments in Fig. 2**b** and Extended Data Fig. 3**a** of the main text. Error bars represent the standard deviation over five rounds of measurements, with each taking 10,000 shots.

Figure S12**b** shows the lifetime of logical and physical Z, Y operators. At $h_x = 0$, the edge modes decay more rapidly than the physical qubits, primarily due to errors from the additional two-qubit gates involved. As h_x increases to 0.11, the physical operators exhibit pronounced oscillations, whereas the edge modes are almost unchanged. Similar behavior is observed for the Bell state fidelity shown in Fig. S12**c**. From an experimental perspective, such perturbations can be modeled as low-frequency noise, where the noise strength remains approximately constant within a single experimental run but fluctuates across runs. To simulate this, we randomly sampled 5 sets of h_x from $[-0.11, 0.11]$ to represent the strength of low-frequency noise in each run, with the averaged values over these runs also displayed in Fig. S12**b**, **c**. Under this noise model, the physical qubits decay significantly faster than the edge modes, clearly demonstrating that the edge modes are more resilient to low-frequency noise than physical qubits.

G. More data for echo experiments

Theoretically, echo dynamics should decay more slowly than the case of $\{|\Psi_0\rangle\}$, serving as an upper bound for edge-mode dynamics when only depolarizing noise exists in the circuit [S27]. However, the observed decay of $\tilde{X}_{\rm L}$ in the echo experiment is slightly faster than that of $\{|\Psi_0\rangle\}$ in our initial experimental runs (see Fig. S13 and Ref. [S28]). As it is hard to identify what exactly happened during the prior echo experiment, we suspect that there were some qubits interfered by unexpected two-level system (TLS) defects that are mobile in the frequency domain (Fluctuating TLS defects have been confirmed in a number of experiments including Ref. [S29]). As a result, the gate parameters could drift slightly yielding extra coherent error during the echo dynamics. We rerun this echo experiment using nominally the same parameters after re-calibrating the experimental system, and obtained the improved results as shown in Fig. 2**b** of the main text.

[S1] Abanin, D. A., De Roeck, W. & Huveneers, F. Exponentially slow heating in periodically driven many-body systems. *Phys. Rev. Lett.* **115**, 256803 (2015).
[S2] Mori, T., Kuwahara, T. & Saito, K. Rigorous bound on energy absorption and generic relaxation in periodically driven quantum systems. *Phys. Rev. Lett.* **116**, 120401 (2016).
[S3] Abanin, D. A., De Roeck, W., Ho, W. W. & Huveneers, F. A rigorous theory of many-body prethermalization for periodically driven and closed quantum systems. *Commun. Math. Phys.* **354**, 809–827 (2017).
[S4] Magnus, W. On the exponential solution of differential equations for a linear operator. *Commun. Pure Appl. Math.* **7**, 649–673 (1954).
[S5] Kuwahara, T., Mori, T. & Saito, K. Floquet–Magnus theory and generic transient dynamics in periodically driven many-body quantum systems. *Ann. Phys.* **367**, 96–124 (2016).
[S6] Heyl, M., Hauke, P. & Zoller, P. Quantum localization bounds Trotter errors in digital quantum simulation. *Sci. Adv.* **5**, eaau8342 (2019).

[S7] Fendley, P. Strong zero modes and eigenstate phase transitions in the XYZ/interacting Majorana chain. *J. Phys. A: Math. Theor.* **49**, 30LT01 (2016).
[S8] Kemp, J., Yao, N. Y., Laumann, C. R. & Fendley, P. Long coherence times for edge spins. *J. Stat. Mech.* **2017**, 063105 (2017).
[S9] Kemp, J., Yao, N. Y. & Laumann, C. R. Symmetry-enhanced boundary qubits at infinite temperature. *Phys. Rev. Lett.* **125**, 200506 (2020).
[S10] Else, D. V., Bauer, B. & Nayak, C. Prethermal phases of matter protected by time-translation symmetry. *Phys. Rev. X* **7**, 011026 (2017).
[S11] Else, D. V., Fendley, P., Kemp, J. & Nayak, C. Prethermal strong zero modes and topological qubits. *Phys. Rev. X* **7**, 041062 (2017).
[S12] Machado, F., Else, D. V., Kahanamoku-Meyer, G. D., Nayak, C. & Yao, N. Y. Long-range prethermal phases of nonequilibrium matter. *Phys. Rev. X* **10**, 011043 (2020).
[S13] Else, D. V., Ho, W. W. & Dumitrescu, P. T. Long-lived interacting phases of matter protected by multiple time-translation symmetries in quasiperiodically driven systems. *Phys. Rev. X* **10**, 021032 (2020).
[S14] Lieb, E., Schultz, T. & Mattis, D. Two soluble models of an antiferromagnetic chain. *Ann. Phys.* **16**, 407–466 (1961).
[S15] Thakurathi, M., Patel, A. A., Sen, D. & Dutta, A. Floquet generation of Majorana end modes and topological invariants. *Phys. Rev. B* **88**, 155133 (2013).
[S16] Akila, M., Waltner, D., Gutkin, B. & Guhr, T. Particle-time duality in the kicked Ising spin chain. *J. Phys. A: Math. Theor.* **49**, 375101 (2016).
[S17] Bertini, B., Kos, P. & Prosen, T. Exact spectral form factor in a minimal model of many-body quantum chaos. *Phys. Rev. Lett.* **121**, 264101 (2018).
[S18] Lerose, A., Sonner, M. & Abanin, D. A. Scaling of temporal entanglement in proximity to integrability. *Phys. Rev. B* **104**, 035137 (2021).
[S19] Orús, R. A practical introduction to tensor networks: Matrix product states and projected entangled pair states. *Annals of Physics* **349**, 117–158 (2014).
[S20] Cirac, J. I., Pérez-García, D., Schuch, N. & Verstraete, F. Matrix product states and projected entangled pair states: Concepts, symmetries, theorems. *Rev. Mod. Phys.* **93**, 045003 (2021).
[S21] Haghshenas, R. *et al.* Digital quantum magnetism at the frontier of classical simulations. *arXiv:2503.20870* (2025).
[S22] Ren, W. *et al.* Experimental quantum adversarial learning with programmable superconducting qubits. *Nat. Comput. Sci.* **2**, 711–717 (2022).
[S23] Neill, C. *et al.* Accurately computing the electronic properties of a quantum ring. *Nature* **594**, 508–512 (2021).
[S24] Mi, X. *et al.* Time-crystalline eigenstate order on a quantum processor. *Nature* **601**, 531–536 (2022).
[S25] Foxen, B. *et al.* Demonstrating a continuous set of two-qubit gates for near-term quantum algorithms. *Phys. Rev. Lett.* **125**, 120504 (2020).
[S26] Smolin, J. A., Gambetta, J. M. & Smith, G. Efficient method for computing the maximum-likelihood quantum state from measurements with additive gaussian noise. *Phys. Rev. Lett.* **108**, 070502 (2012).
[S27] Mi, X. *et al.* Time-crystalline eigenstate order on a quantum processor. *Nature* **601**, 531–536 (2022).
[S28] Jin, F. *et al.* Observation of topological prethermal strong zero modes. *arXiv: 2501.04688* (2025).
[S29] Klimov, P. V. *et al.* Fluctuations of energy-relaxation times in superconducting qubits. *Phys. Rev. Lett.* **121**, 090502 (2018).
